# Metabolic regulation of the carbonyl stress pathway and methylglyoxal in Lyme disease and relapsing fever spirochetes

**DOI:** 10.1371/journal.ppat.1014657

**Published:** 2026-09-29

**Authors:** Dan Drecktrah, Laura S. Hall, Britney Cheff, Crystal Richards, Sandra J. Raffel, Michael Wulf, Ian T. Bailey, Frank Gherardini, D. Scott Samuels

**Affiliations:** 1 Division of Biological and Biomedical Sciences, University of Montana, Missoula, Montana, United States of America; 2 Center for Biomolecular Structure and Dynamics, University of Montana, Missoula, Montana, United States of America; 3 Laboratory of Bacteriology, Rocky Mountain Laboratories, Division of Intramural Research, National Institute of Allergy and Infectious Diseases, National Institutes of Health, Hamilton, Montana, United States of America; Virginia Commonwealth University Medical Center, UNITED STATES OF AMERICA

## Abstract

The carbonyl stress pathway in bacteria is mediated by the enigmatic metabolite methylglyoxal and is thought to be activated in response to cellular carbohydrate and phosphate imbalance. Methylglyoxal, produced from dihydroxyacetone phosphate by methylglyoxal synthase, covalently modifies nucleophilic groups of amino acid residues, such as lysine, arginine and cysteine, to yield advanced glycation end products. In this work, we have identified several environmental signals, including glycerol and peptides, which activate carbonyl stress in the Lyme disease spirochete *Borrelia* (*Borreliella*) *burgdorferi*. We establish that methylglyoxal production and protein glycation is dependent on the *mgsA* gene (*bb0364*) product and show that this pathway is active in Lyme disease spirochetes, but not relapsing fever *Borrelia* or in *B. mayonii*. Furthermore, we have investigated the role of carbonyl stress in the tick-mouse model of Lyme disease and provide evidence that production of methylglyoxal and glycation of proteins is not necessarily lethal, as previously believed, at least in Lyme disease spirochetes. Finally, we show that expression of the *B. burgdorferi mgsA* and carbonyl stress pathway in the relapsing fever spirochete *B. hermsii* significantly reduces the peak level of spirochetemia during the relapse phase of murine infection. Together, our results identify a new post-translation modification system in Lyme disease spirochetes that appears to be important in governing the lifestyle of this pathogen and seems to be selected against in spirochetes that reach high numbers in the blood.

## Author summary

The etiological agents of Lyme disease and relapsing fever are closely related bacteria in the deeply branching spirochete phylum. In North America, Lyme disease is primarily caused by *Borreliella* (formerly *Borrelia*) *burgdorferi* and relapsing fever is predominantly caused by *Borrelia hermsii* and *Borrelia turicatae*. Both groups of spirochetes exist in enzootic cycles involving a tick vector and a vertebrate host; however, *B. burgdorferi* is transmitted slowly (>48 h) by hard ticks in the genus *Ixodes* and persists in the tick midgut, while *B. hermsii* is rapidly transmitted (within minutes) by soft ticks in the genus *Ornithodoros* and resides in the tick salivary glands. In this study, we identify and characterize an unusual and understudied metabolic system, the carbonyl stress pathway, which is present in Lyme disease spirochetes but absent in relapsing fever spirochetes. We found this pathway is induced by external glycerol and peptides, and is mediated by methylglyoxal synthase, which converts dihydroxyacetone phosphate to methylglyoxal. Methylglyoxal is a highly reactive electrophile that modifies proteins to form advanced glycation end products that have been implicated in proinflammatory diseases. We present evidence, both *in vitro* and *in vivo*, that methylglyoxal is not necessarily lethal and that the carbonyl stress pathway controls spirochete density in the host blood during infection.

## Introduction

The causative agent of Lyme disease (LD) in North America, *Borrelia* (*Borreliella*) *burgdorferi*, exists exclusively in either an Ixodid tick or a vertebrate host. The natural enzootic lifecycle begins as naïve tick larvae feed upon an infected host to acquire the spirochete. *B. burgdorferi* persists in the midgut after the blood meal and as the larvae molt into nymphs, the spirochetes can then be transmitted to a new host when the tick takes a second blood meal, thus completing the cycle [1–3]. Available nutrients vary as *B. burgdorferi* progresses through the enzootic cycle, from blood meals rich with glucose to the austere conditions between blood meals in the tick midgut and disseminated locations in the vertebrate such as the synovial fluid in the joints [3,4]. *B. burgdorferi* is particularly dependent on and sensitive to environmental nutrients as it lacks the metabolic pathways for *de novo* amino acid and nucleic acid biosyntheses as well as gluconeogenesis, the citric acid cycle and an electron transport chain [5–7].

Numerous carbon sources such as glucose, mannose, *N*-acetylglucosamine, trehalose, maltose, chitobiose, and glycerol as well as peptides and amino acids, such as glutamate and aspartate, have been identified to serve as energy sources for *B. burgdorferi* growth [8–11]. Glucose is thought to be the preferred carbon source and is likely more important in the vertebrate host and during blood meals, while glycerol is important to support survival in the tick and as a signal to regulate expression of the glycerol (*glp*) metabolic operon [12–15]. The *glp* operon consists of genes encoding a glycerol transport facilitator (*glpF*), a glycerol kinase (*glpK*) and a glycerol-3-phosphate dehydrogenase (*glpD*), where the gene products are responsible for the movement of glycerol across the inner membrane, phosphorylation of glycerol to glycerol-3-phosphate (G3P) and conversion of G3P to dihydroxyacetone phosphate (DHAP), respectively [4–6]. Metabolic control of glycerol appears to be crucial in the spirochete as the *glp* operon is regulated by two global intracellular second messengers, as well as other transcriptional regulators: the stringent response mediated by guanosine tetra- and pentaphosphate ((p)ppGpp) produced and hydrolyzed by Rel_Bbu_ and the cyclic-di-GMP (c-di-GMP) signaling pathway mediated by the response regulator 1 (Rrp1) [13–17]. Interestingly, both signaling pathways are important for *B. burgdorferi* in the tick, where glycerol is known to play a role in persistence [12,14,18]: Rel_Bbu_ is important for survival and persistence in the unfed nymph [16] and Rrp1 is crucial during tick acquisition and persistence [14,19,20]. Moreover, the key regulators RpoS (an alternative sigma factor that controls expression of genes required for vertebrate infection) [13,21], BosR (an activator of RpoS production) [22,23], BadR (a transcription factor) [24], HrpA (a DEAH-box RNA helicase) [25], SpoVG (a nucleic acid-binding protein) [26], DksA (a cofactor of the stringent response) [27], and PlzA (the c-di-GMP-binding protein) [28] all regulate *glp* expression, underscoring the central role of glycerol metabolism in the spirochete. Furthermore, we have recently identified that GlpD, along with GpsA, define a crucial metabolic lynchpin that regulates NADH and G3P levels and is essential for persistence in the tick, again highlighting the importance of this seemingly simple metabolic pathway [18]. Curiously, under nutrient stress conditions *in vitro*, we found that glycerol can be cytotoxic to *B. burgdorferi*, likely inducing metabolic imbalances and perhaps affecting the NAD^+^/NADH redox potential, resulting in cell death by an unknown mechanism(s) [18]. Thus, the metabolic response to glycerol and the intricate regulation of its operon suggest that this system is fundamental to defining *B. burgdorferi*’s lifestyle in ticks with implications regarding both transmission to and acquisition from vertebrate hosts.

The carbonyl stress pathway (CSP), a signaling system found only in bacteria, is triggered by metabolic imbalances, excess glucose and oxidative stress; the pathway is mediated by the enigmatic three-carbon metabolite methylglyoxal (MG) [29–31]. The CSP and the existence of MG in cells have been known for over 100 years, but the function of this reactive electrophile remains largely unknown [32]. In bacteria, methylglyoxal synthase (MgsA) converts the glycolytic intermediate DHAP to MG and inorganic phosphate [33] (Fig 1A). Certain conditions of carbohydrate imbalance induce MG production in *Escherichia coli* [30]. This pathway may serve to bypass the lower half of glycolysis in order to regenerate inorganic phosphate necessary for cell survival [29]. Accumulation of MG must be tightly regulated as this intermediate is highly reactive and covalently modifies the amino groups of proteins forming advanced glycation end products (AGEs) [34], becoming cytotoxic as levels increase [35]. AGEs have been implicated in many human diseases including heart disease, cancer, diabetes, and other inflammatory conditions, but have not been well studied in pathogenic bacteria and their associated diseases [36–38]. So, while MG can be chemically produced by oxidation of sugars in some capacity in all organisms, no eukaryotes are known to possess an MgsA ortholog [39], making the CSP a potential therapeutic target for LD prevention or treatment.

**Fig 1:**
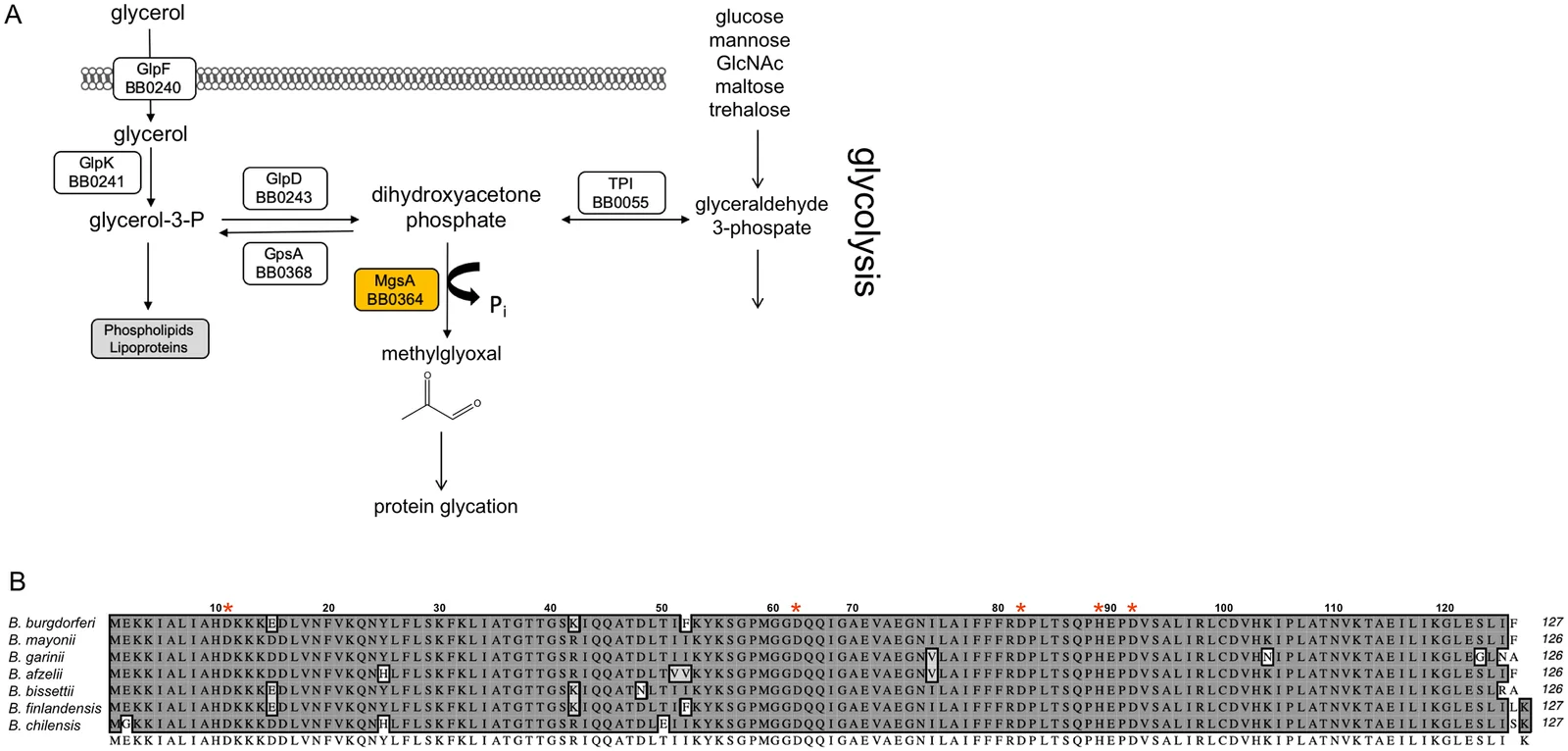
Phylogenetic tree of MgsA protein sequences. (A) Carbonyl stress pathway showing methylglyoxal synthase (MgsA), which converts dihydroxyacetone phosphate to methylglyoxal and inorganic phosphate (P_*i*_), located at the node connecting glycolysis and phospholipid and lipoprotein biosynthesis. (B) Clustal alignment of *Borrelia* (*Borreliella*) MgsA protein sequences. Active site residues are marked with red asterisks.

MgsA is nearly ubiquitous among bacteria. All LD spirochetes whose genomes have been sequenced possess an annotated *mgsA* gene, but relapsing fever (RF) spirochete genomes lack an *mgsA* ortholog (Table 1). In this work we demonstrate that glycerol and neopeptone induce the CSP in LD spirochetes, establish that the CSP depends on the *mgsA* gene, and examine the role of the CSP in the tick-mouse model of LD. Furthermore, we show that heterologously expressing the LD spirochete *mgsA* gene in RF spirochetes attenuates host infectivity using the mouse model of RF, which is the first example, to our knowledge, of a *B. burgdorferi* gene expressed and functioning in an RF *Borrelia*.

**Table 1:** Distribution of glycerol and methylglyoxal metabolic genes in LD and RF spirochete species.

	vector	species	*glp* *FKD*	*glpQ*	*glpT*	*mgsA*
**LD**	*Ixodes*	*B.* *burgdorferi*	✓	–	–	✓
	*Ixodes*	*B.* *garinii*	✓	–	–	✓
	*Ixodes*	*B.* *afzelii*	✓	–	–	✓
	*Ixodes*	*B.* *mayonii*	✓	–	–	✓
**RF**	*Ixodes*	*B.* *miyamotoi*	✓	✓	✓	–
	*Ornithodoros*	*B.* *hermsii*	✓	✓	✓	–
	*Ornithodoros*	*B.* *turicatae*	✓	✓	✓	–
	*Ornithodoros*	*B.* *parkeri*	✓	✓	✓	–
	*Ornithodoros*	*B.* *duttoni*	✓	✓	✓	–
	*Ornithodoros*	*B.* *crocidurae*	✓	✓	✓	–
	*Pediculus* *humanus*	*B.* *recurrentis*	*glpF* only	✓	✓	–

## Results

The CSP enzymatically produces MG from the intermediate metabolite DHAP via the action of MgsA (Fig 1A). *mgsA* is found in the genomes of almost all bacteria but is absent from eukaryotes. The LD agent *B. burgdorferi* has a predicted *mgsA* gene (*bb0364*) located on the chromosome [5]. The *mgsA* gene of LD spirochetes from the Americas and Eurasia all contain the conserved amino acid residues predicted to be in the active site [40] (Fig 1B, red asterisks) and share ≥96% identity.

To determine if the CSP was active in *B. burgdorferi* and identify the signals for induction, we grew spirochetes in normal growth medium (BSK + RS) at 35°C to late log phase before collecting cells by centrifugation and resuspending cultures in nutrient stress medium containing various carbohydrates or other media components as we have previously described [18]. After 24 h, total cell lysates were collected and analyzed by immunoblotting using antibodies against MG that recognize glycated proteins or antibodies against FlaB as a loading control. Increasing concentrations of glycerol in nutrient stress medium induced the CSP, resulting in numerous glycated proteins as illustrated by a constellation of bands (Fig 2A, glycerol). Adding MG directly to the medium of cells resulted in a similar set of glycated proteins (Fig 2A, MG). Further screening revealed that incubating *B. burgdorferi* in nutrient stress medium containing 0.1% neopeptone, an enzymatic digest of casein protein, or normal BSK growth medium lacking bovine serum albumin (BSA) and rabbit serum (RS) also induced the CSP and protein glycation (Fig 2B). No other assayed sugars, including mannose, maltose, trehalose or *N*-acetyl glucosamine, induced the CSP (Fig 2C). Interestingly, adding BSA but not RS blocked glycerol-induced glycation of *B. burgdorferi* proteins (Fig 2C).

**Fig 2:**
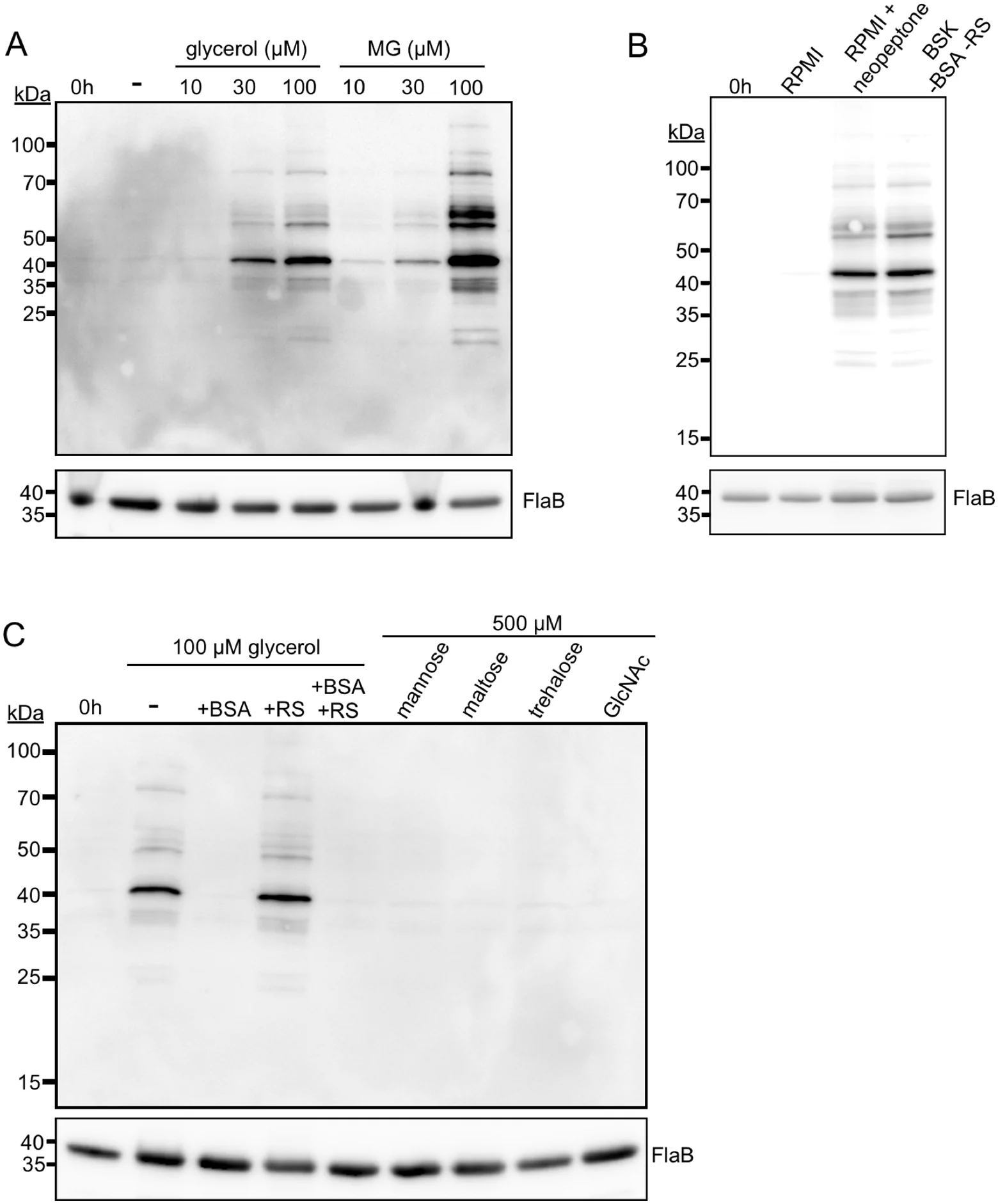
Glycerol induces the CSP and glycation in *B. burgdorferi.* (A) *B. burgdorferi* were grown to ~7 × 10^7^ cells ml^−1^ before collection by centrifugation (0h) and resuspension in RPMI (-), RPMI with 10, 30 or 100 µM glycerol or 10, 30 or 100 µM methylglyoxal (MG) and incubation for 24 h at 35°C. Equal amounts of whole cell lysates were separated by SDS-PAGE and visualized by immunoblot analyses with anti-MG antibodies to detect protein glycation and anti-FlaB antibodies as a loading control. (B) Cells were grown as in panel A but were incubated in RPMI + 0.4% neopeptone or BSK without bovine serum albumin or rabbit serum (BSK -BSA -RS) for 24 h at 35°C. (C) Cells were grown as in panel A but were incubated in RPMI + 100 µM glycerol alone (-) or containing BSA (+BSA), RS (+RS) or both (+BSA + RS) for 24 h at 35°C. Also, cells were incubated in RPMI containing 500 µM mannose, maltose, trehalose or *N*-acetyl glucosamine (GlcNAc) for 24 h at 35°C. Cell lysates were analyzed by immunoblot as described in panel A.

To investigate if the CSP is dependent on the predicted *mgsA* gene (*bb0364*) and if MgsA is responsible for protein glycation, we generated a null *mgsA* mutant by replacing the *mgsA* open reading frame with an antibiotic resistance cassette (∆*mgsA*) (Fig 3A). The *mgsA cis*-complemented strain (*mgsA*^+^) was constructed by restoring the *mgsA* gene in its native locus on the chromosome (Fig 3A). MgsA production in the wild-type (WT), ∆*mgsA* and *mgsA*^+^ strains was verified by immunoblot of whole cell lysates using antibodies against *B. burgdorferi* MgsA protein (predicted to be ~ 14 kDa). MgsA was present in WT, absent in the ∆*mgsA* mutant and restored in the complemented strain (Fig 3B). To test if the CSP was dependent on the *mgsA* gene, WT, ∆*mgsA* and *mgsA*^+^ strains were grown in normal growth medium before shifting to nutrient stress medium alone (RPMI) or containing glycerol (+ 100 µM glycerol) for 24 h; cell lysates were collected and analyzed by immunoblot with anti-MG antibodies. Glycerol induced the CSP and glycation in WT (as seen in Fig 2) and *mgsA*^+^ strains, while glycation of proteins was absent in the ∆*mgsA* strain (Fig 3C), demonstrating that *mgsA* is required for the CSP in *B. burgdorferi*. The requirement for *mgsA* in the CSP was not limited to induction by glycerol as both neopeptone and removal of BSA and RS both induced the CSP in an *mgsA*-dependent fashion (Fig 3D). Externally added MG successfully glycated the proteins from all strains, indicating that proteins from the ∆*mgsA* strain can be glycated, but enzymatic glycation depends on *mgsA* (Fig 3C). To examine if glycerol-induced glycation and activation of the CSP depends on the import of glycerol and its enzymatic conversion to DHAP, we removed the ability of *B. burgdorferi* to transport glycerol into the cell and metabolize glycerol by generating null mutants in the glycerol uptake facilitator (∆*glpF*) and glycerol kinase (∆*glpK*). We tested these mutants, as well as our extant glycerol-3-phosphate dehydrogenase (∆*glpD*) mutant [18], and their respective complemented strains, for their ability to glycate proteins in response to added glycerol. All three *glp* mutants were unable to glycate proteins in response to glycerol and glycation was restored in the complemented strains (Fig 4). Thus, glycerol must be transported into the spirochete, phosphorylated to glycerol-3-phosphate and converted to DHAP to activate the CSP.

**Fig 3:**
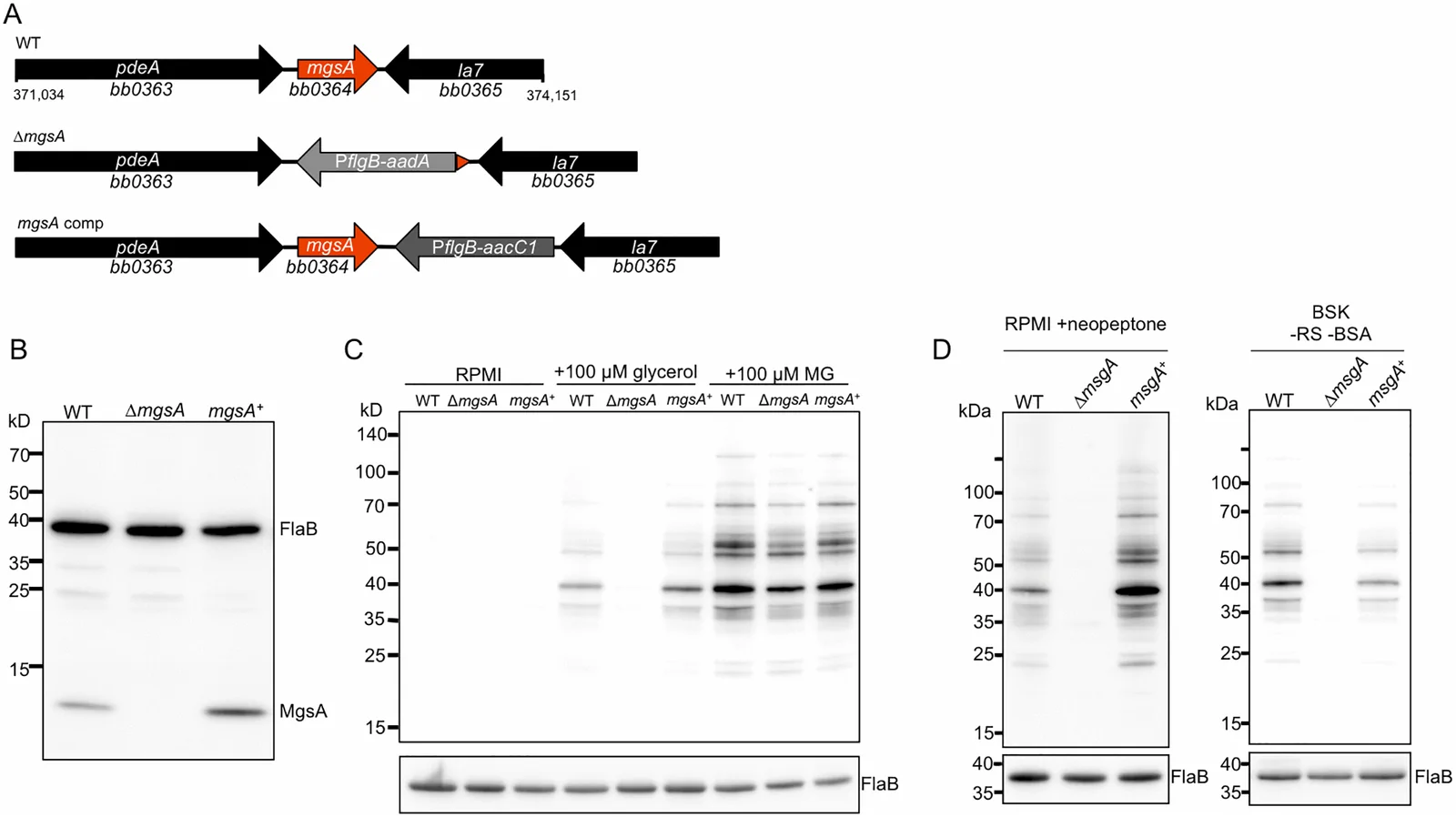
Genetic disruption of the *mgsA* gene (*bb0364*). (A) The location of the predicted *mgsA* gene on the chromosome of *B. burgdorferi* B31 is illustrated (nucleotide number references based on NCBI Ref Seq: NC_001318.1) and graphic representation of the *mgsA* mutant (∆*mgsA*) and *mgsA* complemented (*mgsA*^+^) strains. (B) Immunoblot of total cell extracts from wild-type (WT), ∆*mgsA* and *mgsA*^+^ strains using anti-MgsA and anti-FlaB antibodies (loading control). (C) Anti-MG immunoblot of glycated proteins (upper panel) from total cell extracts of wild-type (WT), ∆*mgsA* and *mgsA*^+^ strains in RPMI (nutrient stress), RPMI with 100 µM glycerol or 100 µM methylglyoxal (MG) for 24 h at 35°C. (D) Anti-MG immunoblot of glycated proteins (upper panel) from total cell extracts of wild-type (WT), ∆*mgsA* and *mgsA*^+^ strains in RPMI with 0.4% neopeptone or BSK without BSA and without RS (BSK -BSA -RS) for 24 h at 35°C.

**Fig 4:**
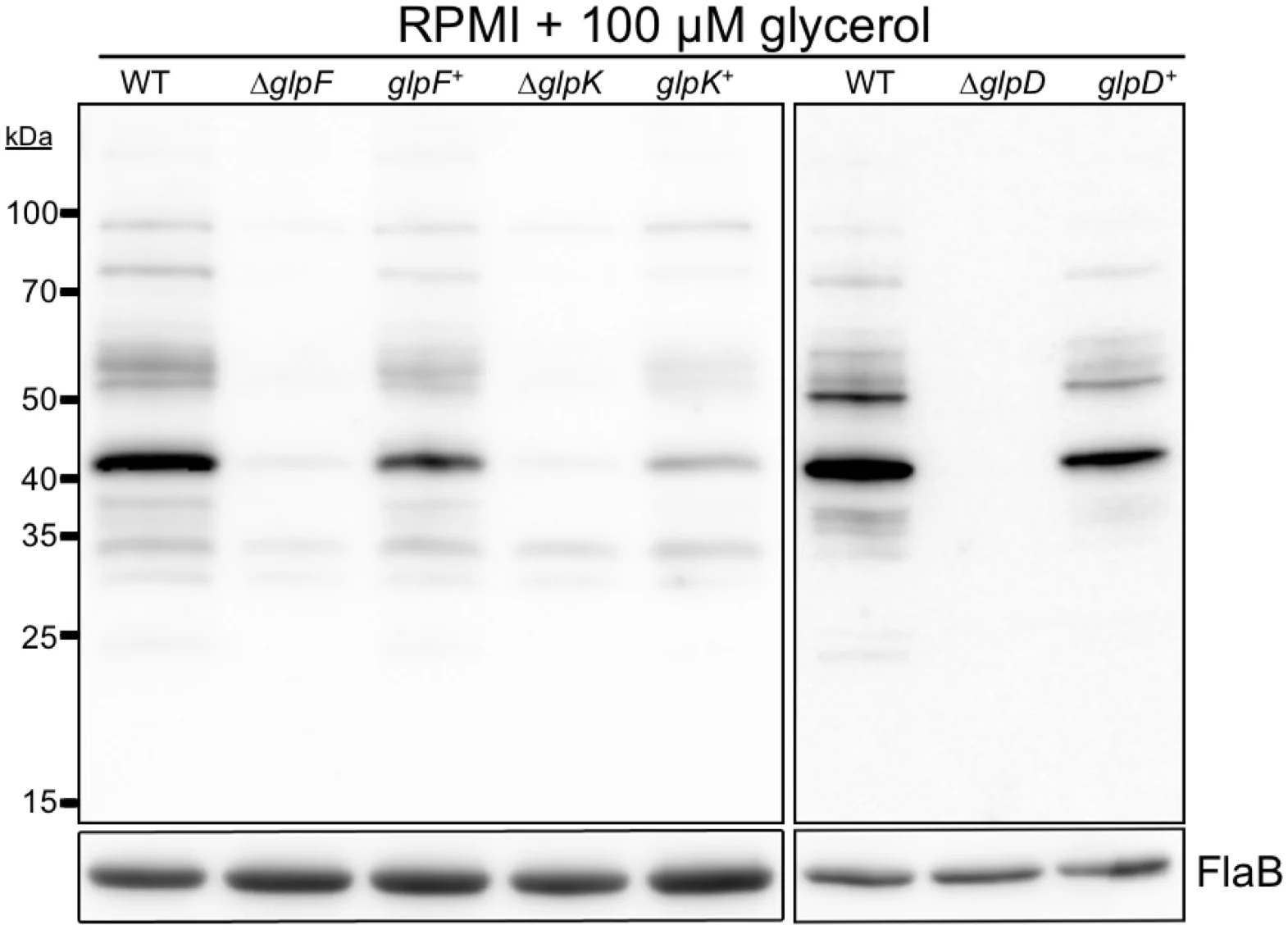
Glycerol import and metabolism are required to induce the CSP. *B. burgdorferi* wild-type (WT), *glpF* null mutant (*∆glpF*), *glpF* complement (*glpF*^+^), *glpK* null mutant (*∆glpK*), *glpK* complement (*glpK*^+^), *glpD* null mutant (*∆glpD*), and *glpD* complement (*glpD*^+^) strains were grown to ~7 × 10^7^ cells ml^−1^ before collection by centrifugation, resuspension in RPMI + 100 µM glycerol and incubation for 24 h at 35°C. Equal amounts of whole cell lysates were separated by SDS-PAGE and analyzed by immunoblot with anti-MG antibodies to detect protein glycation and anti-FlaB antibodies as a loading control.

Induction of the CSP leading to MG production is thought to be lethal in bacteria [29,41], but the effect on LD spirochetes has not been examined. To compare how induction of the CSP and glycation by glycerol or glycation by externally added MG affect the viability of *B. burgdorferi*, spirochetes were exposed to increasing concentrations of glycerol or MG in RPMI and then plated in semi-solid growth media. After growth, colonies were enumerated and the percent survival was expressed relative to the number of colonies before exposure to RPMI, glycerol or MG as we have previously described [18]. *B. burgdorferi* viability drops significantly in 40 µM glycerol compared to RPMI alone and cells were not viable in 100 µM glycerol (Fig 5A), confirming our previously reported cytotoxicity [18]. Surprisingly, 100 µM MG was not cytotoxic to cells as they survived as well as those in nutrient stress media alone (Fig 5B, compare 0 µM to 100 µM MG), suggesting that even though proteins are glycated (see Fig 2A and 3C), cell viability was not affected. Higher concentrations of MG significantly reduced *B. burgdorferi* viability (Fig 5B). To examine if the glycerol-induced cytotoxicity was dependent on the carbonyl stress pathway, the viability of the ∆*mgsA* mutant was measured after incubation in 100 µM glycerol in RPMI as described above. The ∆*mgsA* strain remained viable in 100 µM glycerol and survived significantly better than both wild-type and *mgsA*^+^ strains in glycerol (Fig 5C), suggesting that the carbonyl stress pathway is responsible for susceptibility to glycerol.

**Fig 5:**
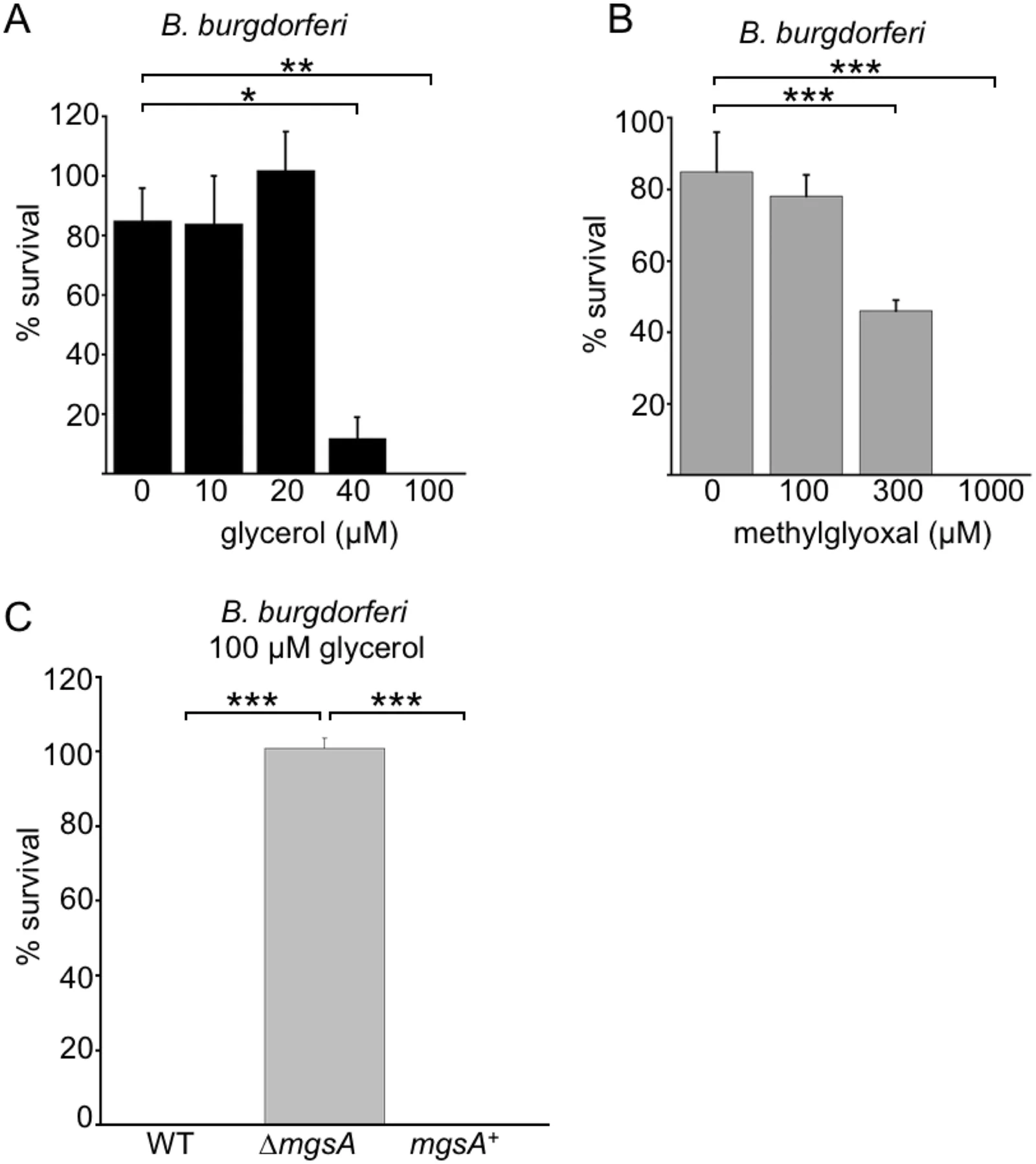
Viability of the LD spirochete *B. burgdorferi* in glycerol and MG. Wild-type *B. burgdorferi* were grown in normal growth medium before shifting to RPMI medium alone or RPMI containing increasing concentrations of glycerol (A) or MG (B) for 24 h at 35°C. Cells were plated in semi-solid BSK before and after shifting to RPMI alone (0) or with increasing concentrations of glycerol or MG. (C) Wild-type (WT), *mgsA* mutant (∆*mgsA*) and *mgsA*-complemented (*mgsA*^+^) strains were grown as described in panel A, shifted to RPMI medium containing 100 µM glycerol for 24 h at 35 °C and then plated. Values are the mean of three independent biological replicates expressed as % survival compared to cultures before shifting to RPMI. Error bars are the SE of the mean. Asterisks represent: * *P* = 0.006, ** *P* = 0.002 and *** *P* < 0.001 as determined by one-way ANOVA with a Tukey’s *post-hoc* test.

Next, we analyzed the expression of MgsA during *B. burgdorferi* growth in culture at 35°C. MgsA levels were largely independent of cell density from mid-log (1 – 3 × 10^7^ cells ml^−1^) to late stationary phase (~ 3 × 10^8^ cells ml^−1^) (Fig 6A). To examine if glycation depends on growth phase (cell density), the CSP was induced by adding glycerol in RPMI to cells in mid-log, late-log (3 – 7 × 10^7^ cells ml^−1^) and late stationary phase. The CSP and glycation were induced in cells in late-log phase, but not in mid-log or late stationary phase (Fig 6B), suggesting the cellular metabolic state plays a role in CSP induction. Some glycation was observed in all cells in late stationary phase (Fig 6B).

**Fig 6:**
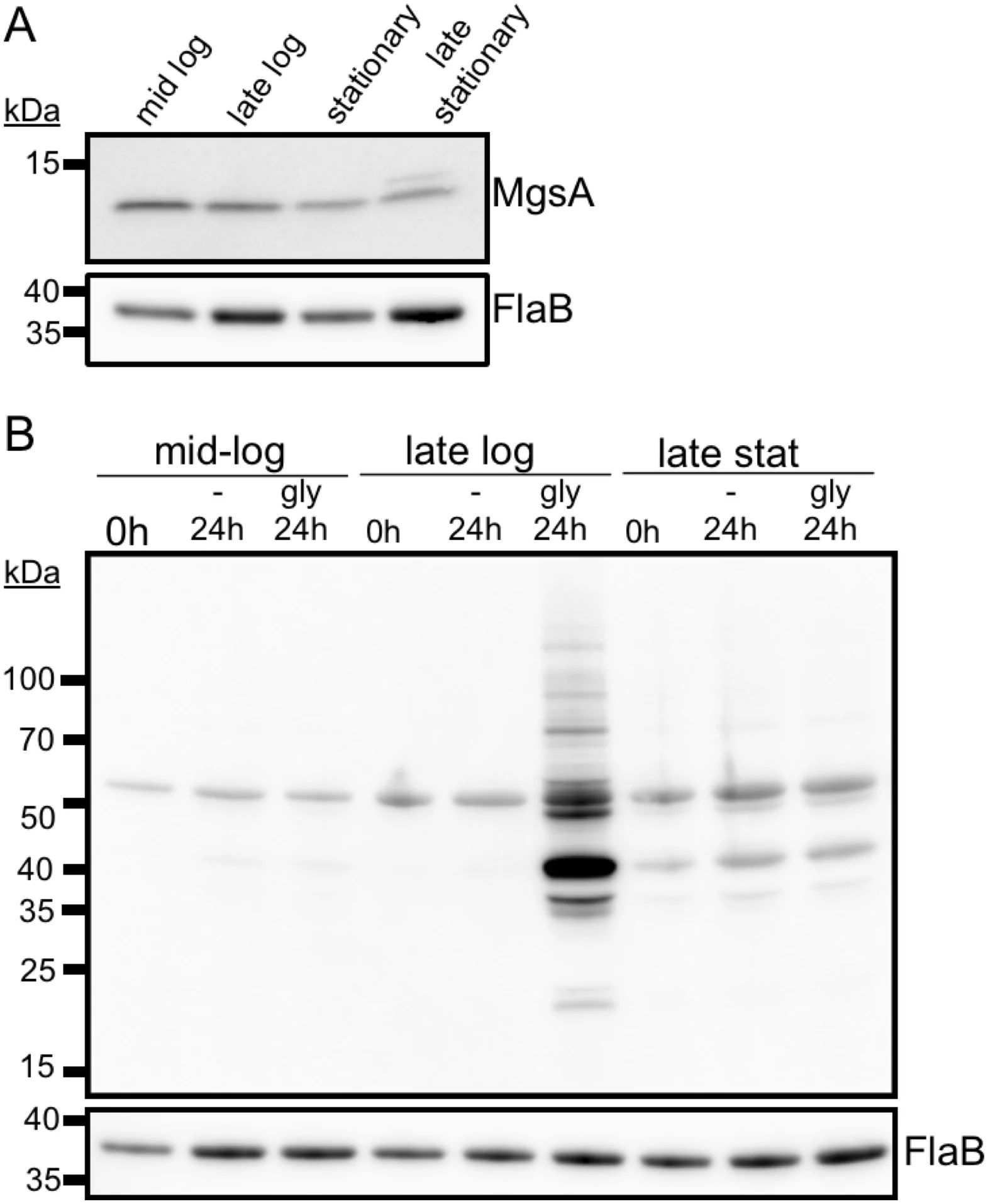
Growth phase affects induction of the CSP but not MgsA levels. (A) *B. burgdorferi* were grown at 35°C until mid-log (~1 × 10^7^ cells ml^−1^), late log (5-7 × 10^7^ cells ml^−1^), stationary (~1 × 10^8^ cells ml^−1^) or late stationary (~3 × 10^8^ cells ml^−1^) phase before whole cell lysates collected. Samples were separated by SDS-PAGE and analyzed by immunoblot using antibodies against MgsA or FlaB. (B) Cells were grown to mid-log, late-log or late stationary phase and total cell lysates collected (0h) or shifted to RPMI (- 24 h) or RPMI containing 100 µM glycerol (gly 24 h) at 35°C. After 24 h, whole cell lysates were collected and analyzed by immunoblot using anti-MG antibodies to follow protein glycation and anti-FlaB antibodies as a loading control.

Intracellular second messengers are crucial for *B. burgdorferi* to adapt to transiting between vector and host, including but not limited to the regulation of metabolic pathways [3,4,42,43]. To examine the role(s) of two key second messengers, (p)ppGpp and c-di-GMP, in controlling the CSP, we followed protein glycation in mutant strains unable to produce (p)ppGpp (∆*rel*_Bbu_) or c-di-GMP (∆*rrp1*) as well as in strains lacking the associated cofactors/effectors DksA (∆*dksA*) and PlzA (∆*plzA*), respectively. The glycerol-induced CSP was partially dependent on Rel_Bbu_ as the ∆*rel* mutant showed less glycation than the wild-type and the *rel*-complemented strains, but the effect of neopeptone-induced glycation was not as dramatic (Fig 7A). The CSP induction was not influenced by the stringent response accessory protein DksA as the ∆*dksA* mutant had similar glycation profiles to that of the wild-type and complemented strains in the presence of glycerol or neopeptone (Fig 7B). The levels of MgsA protein were independent of both the stringent response and c-di-GMP signaling pathways; the reason MgsA appears as a doublet in some instances but not others is not clear (Fig 7): it could be nonspecific binding of the antibodies or possible protein modifications of MgsA.

**Fig 7:**
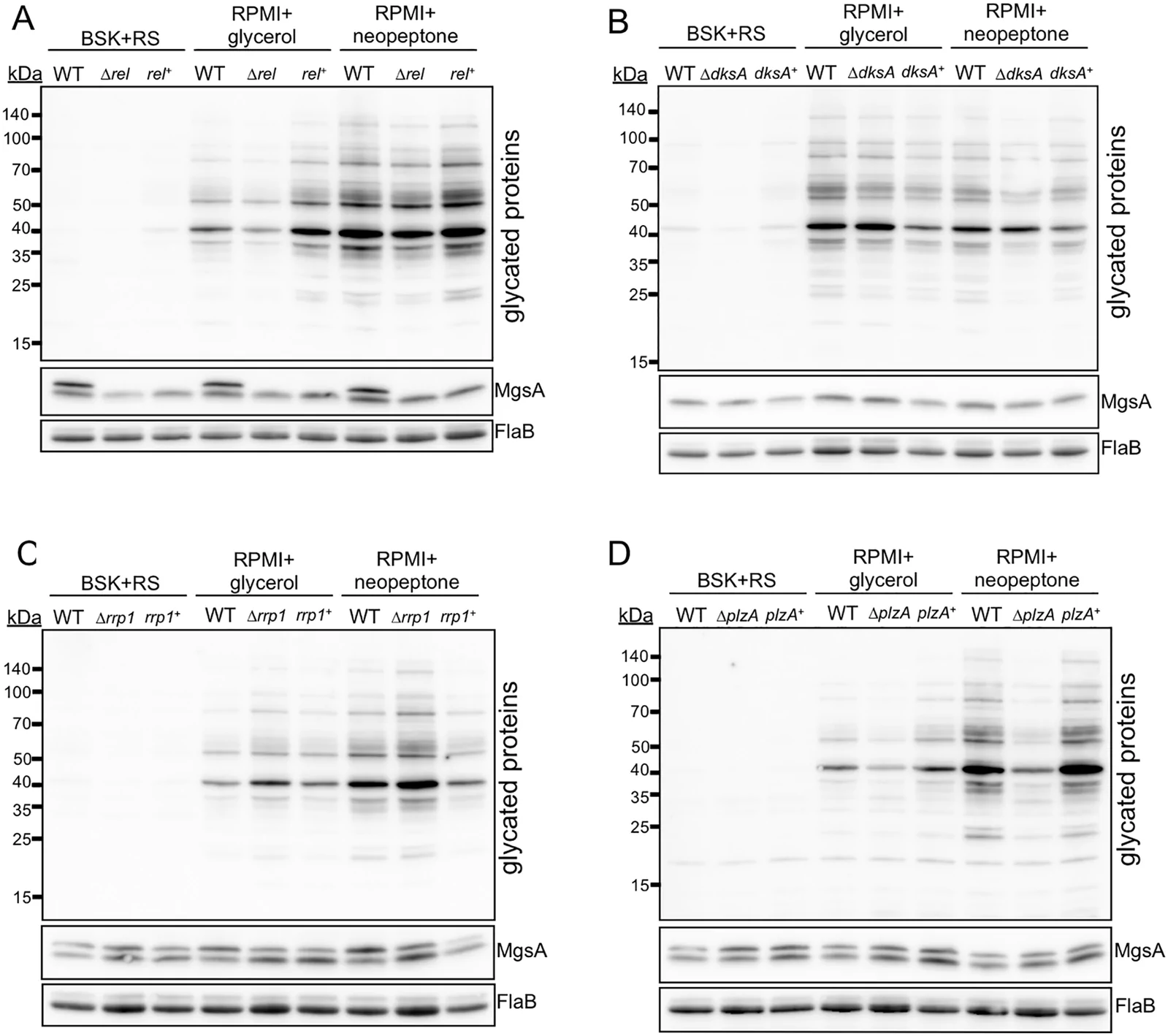
Regulation of the CSP and glycation by the stringent response and c-di-GMP signaling pathways. Wild-type (WT), *rel*_Bbu_ mutant (∆*rel*_Bbu_) (A), *dksA* mutant (∆*dksA*) (B), *rrp1* mutant (∆*rrp1*) (C), or *plzA* mutant (∆*plzA*) (D) and respective complemented strains were grown to ~7 × 10^7^ cell ml^−1^ in normal BSK + RS growth medium and whole cell lysates collected or shifted to nutrient stress medium containing glycerol (RPMI + glycerol) or neopeptone (RPMI + neopeptone) for 24 h before collecting cell lysates. Samples were separated by SDS-PAGE and analyzed by immunoblot using antibodies against MG (to recognize glycated proteins), MgsA and FlaB.

In the *rrp1* mutant strain (∆*rrp1*), which is unable to produce c-di-GMP, increased protein glycation was observed in response to both glycerol and neopeptone (Fig 7C), suggesting that this second messenger represses the CSP. Conversely, in the strain lacking the c-di-GMP-binding protein PlzA (∆*plzA*), glycation was reduced compared to that seen in the wild-type and *plzA* complemented strains when stimulated by either glycerol or neopeptone (Fig 7D). Thus, if c-di-GMP is acting solely through PlzA, as hypothesized, [44–48] then holo-PlzA represses glycation (higher glycation in the *rrp1* null mutant that does not produce c-di-GMP, Fig 7C) while apo-PlzA increases glycation (lower glycation in the *plzA* null mutant, Fig 7D).

We hypothesized that the CSP has a role(s) *in vivo* during persistence in the tick and, possibly, in other environments with a relatively low nutrient availability for *B. burgdorferi*, such as the synovial spaces of joints where the spirochete resides. To examine the role of the CSP *in vivo*, we used the tick-mouse laboratory model of Lyme disease and intradermally injected 10^3^ or 10^4^ cells of wild-type, ∆*mgsA* or *mgsA* complemented strains into C3H-HeJ mice. Infection and dissemination were evaluated at three weeks and five weeks post-inoculation by culturing spirochetes from ear biopsies and bladder or ankle tissue, respectively. There was a defect in infectivity of the *mgsA* mutant strain compared to the wild-type and complemented strains at a dose of 10^3^ cells, but all mice from all three strains were infected when 10^4^ cells were injected (Table 2).

**Table 2:** Infectivity of the ∆*mgsA* mutant by needle inoculation.

			3 weeks		5 weeks	
Route	Cells injected	Strain	Ear	Ear	Ankle	Bladder
**Needle^a^**	1 × 10^3^	WT	3/4	3/4	3/4	3/4
	1 × 10^3^	*∆mgsA*	1/4	1/4	1/4	1/4
	1 × 10^3^	*mgsA* ^+^	3/3	3/3	3/3	3/3
**Needle^a^**	1 × 10^4^	WT	7/7	7/7	7/7	7/7
	1 × 10^4^	*∆mgsA*	7/7	7/7	7/7	7/7
	1 × 10^4^	*mgsA* ^+^	7/7	7/7	7/7	7/7

^a^Intradermal injection.

Because the utilization of glycerol has been shown to be important for *B. burgdorferi* persistence in ticks [12,14,18] and we found that glycerol induces the CSP (Fig 2), we examined the role of *mgsA* and the CSP in persistence in and transmission from ticks. Naïve *Ixodes scapularis* larvae were fed to repletion on mice infected with wild-type, ∆*mgsA* or *mgsA* complement strains. Ticks were allowed to molt into nymphs (approximately 10 weeks) before unfed nymphs were allowed to feed to repletion on naïve mice (three nymphs per mouse). Persistence of *B. burgdorferi* through the tick life stages was followed by qPCR to determine the spirochete load per tick. We found no difference in the levels of wild-type, *mgsA* mutant or complemented strains throughout the tick stages in fed larvae (Fig 8A), unfed nymphs (Fig 8B) and fed nymphs (Fig 8C), suggesting *mgsA* is not important for persistence in the tick, at least in the mouse model. In conjunction with our persistence studies, we also tested if transmission from infected nymphs to mice was *mgsA*-dependent. We found that transmission, infection and dissemination to the ear, ankle and bladder tissues did not require *mgsA*, as all mice were infected by ticks carrying the *mgsA* mutant (Table 3).

**Table 3:** Transmission of the ∆*mgsA* mutant to naïve mice via tick bite.

		3 weeks	5 weeks		
Route	Strain	Ear	Ear	Ankle	Bladder
**Nymph** **bite**^**a**^	WT	5/6	5/6	5/6	5/6
	*∆* *mgsA*	6/6	6/6	6/6	6/6
	*mgsA* ^+^	5/6	5/6	5/6	5/6

^a^3 nymphs/mouse.

**Fig 8:**
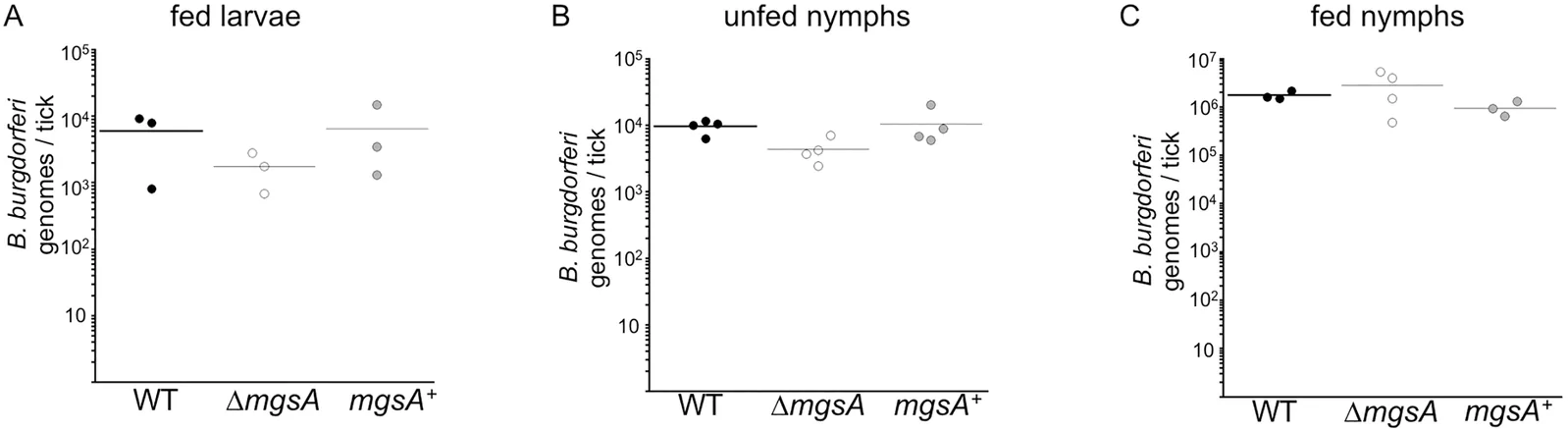
Persistence of the *mgsA* mutant in ticks. *B. burgdorferi* strains were introduced to larval ticks by feeding on WT, ∆*mgsA* or *mgsA*^+^-infected mice. Acquisition and persistence of WT (black circles), ∆*mgsA* mutant (white circles), *mgsA*^+^ (dark gray circles) was assessed in ticks one week after larvae fed to repletion (A), following the molt to nymphs, nine weeks later (B) and one week after nymphs fed to repletion on naïve mice (C). DNA from infected ticks was isolated at each stage and *B. burgdorferi* load in ticks measured by TaqMan qPCR using primers/probe to *flaB*. Bars represent the mean. There was no significant difference between the means as determined by one-way ANOVA with a Tukey’s *post-hoc* test.

We next examined the CSP in *B. mayonii*, an unusual and more recently characterized LD spirochete species that causes different symptoms than *B. burgdorferi* in human infection, including spirochetemia, dispersed rashes and persistent fever [49]. As seen in Table 1, the *B. mayonii* genome, like other LD spirochetes, contains a predicted *mgsA* gene [50]. The CSP was not induced and proteins were not glycated when *B. mayonii* was exposed to glycerol up to 2000 µM, which is twentyfold higher than the concentration that induces the CSP in *B. burgdorferi*. No other sugars tested, including mannose, maltose, trehalose, or *N*-acetylglucosamine, induced the CSP in *B. mayonii* (Fig 9A). Next, we compared the levels of MgsA protein in *B. mayonii* and *B. burgdorferi* by immunoblot. We found that *B. mayonii* expressed much lower levels of MgsA compared to *B. burgdorferi* (Fig 9B), which may explain the lack of glycation in this strain. Thus, even though all LD spirochetes have a predicted *mgsA* gene, expression levels and glycerol-mediated induction of the CSP varies between strains, characteristics that may be a factor responsible for the differing pathologies observed between these LD spirochete species.

**Fig 9:**
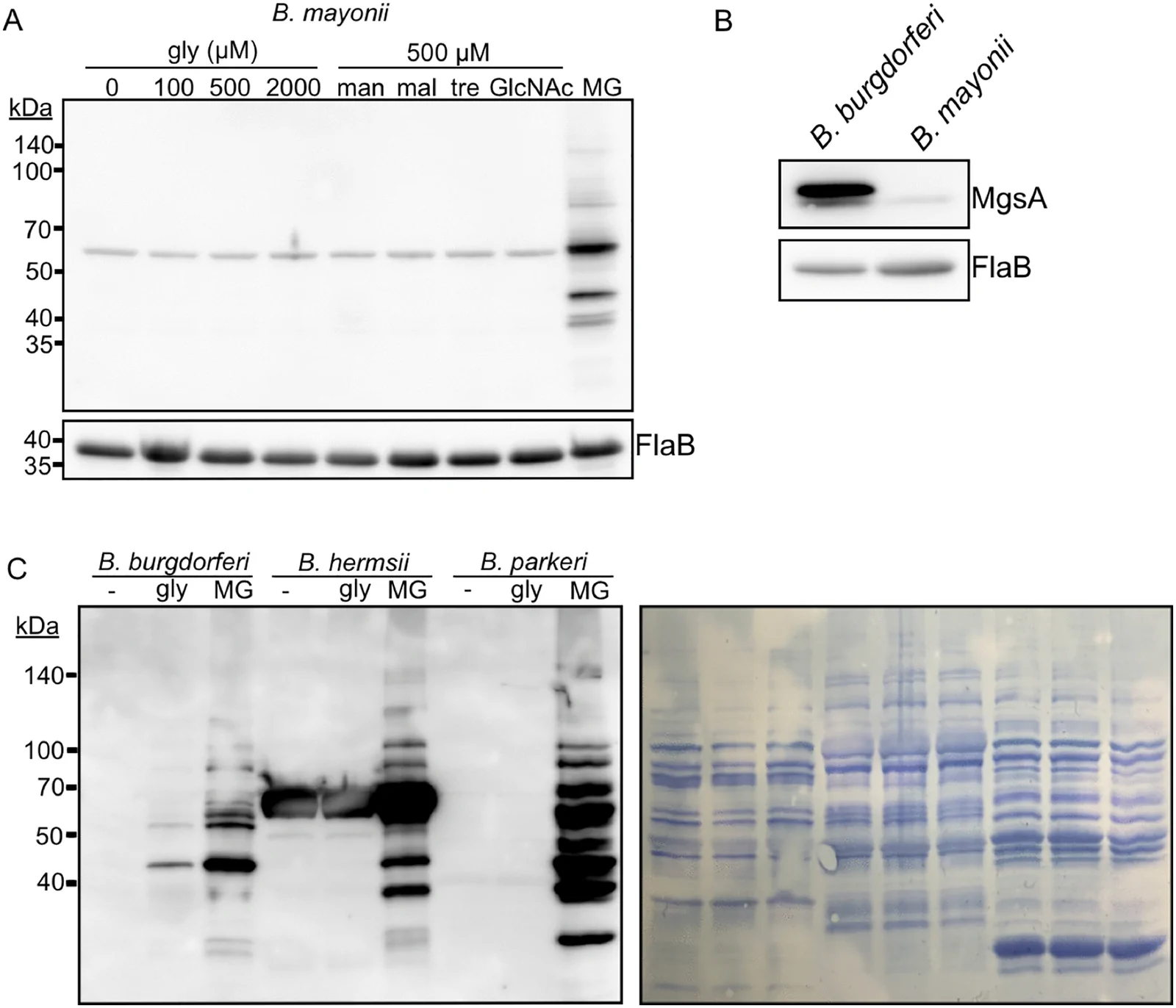
Lack of glycation in the LD spirochete *B. mayonii* and RF *Borrelia* spp. (A) *B. mayonii* was grown to ~7 × 10^7^ cell ml^−1^ before collection by centrifugation and resuspension in RPMI (0), RPMI with 100, 500 or 2000 µM glycerol or 500 µM mannose (man), maltose (malt), trehalose (tre) or *N*-acetyl glucosamine (GlcNAc) or 100 µM methylglyoxal (MG) and incubation for 24 h at 35°C. Equal amounts of whole cell lysates were separated by SDS-PAGE and glycation was visualized by immunoblot analyses with anti-MG antibodies; anti-FlaB antibodies were used as a loading control. (B) Whole cell lysates of *B. burgdorferi* and *B. mayonii* grown in BSK + RS were separated by SDS-PAGE and MgsA levels were visualized by immunoblot analyses with anti-MgsA antibodies; anti-FlaB antibodies were used as a loading control. (C) *B. burgdorferi*, *B. hermsii* and *B. parkeri* were grown to ~7 × 10^7^ cell ml^−1^ before collection by centrifugation and resuspension in RPMI (-), RPMI with 100 µM glycerol (gly) or 100 µM methylglyoxal (MG) and incubation for 24 h at 35°C. Equal amounts of whole cell lysates were separated by SDS-PAGE and protein glycation was measured by immunoblot analyses with anti-MG antibodies. A Coomassie brilliant blue-stained gel is shown as a loading control in the adjacent panel.

RF *Borrelia* are one of the few genera of bacteria that lack a predicted *mgsA* gene (Table 1) [51,52]. To examine how this group of spirochetes responds to external cues that induce the CSP in LD spirochetes, *B. hermsii* and *B. parkeri*, causative agents of tick-borne RF, were exposed to CSP-inducing signals and glycation was analyzed by immunoblot. *B. burgdorferi* exposed to glycerol in nutrient stress media induced protein glycation, but neither *B. hermsii* nor *B. parkeri* showed an induction of protein glycation in response to glycerol, supporting our hypothesis that these spirochetes lack a CSP, or at least respond differently than *B. burgdorferi* (Fig 9C, compare – and gly lanes). There is a notable cross-reactive band around 60 kDa, of unknown identity, present in *B. hermsii* under all conditions, independent of glycation conditions. The proteins of the RF spirochetes could be glycated by adding MG directly to the media (Fig 9C, MG lanes).

Next, we examined the effect of glycerol and MG on the viability of RF *Borrelia*. We hypothesized that *B. hermsii* would be less sensitive to glycerol and likely more sensitive to MG as the RF *Borrelia* lack a predicted *mgsA* gene and CSP. Surprisingly, *B. hermsii* did not persist well in nutrient stress medium alone (RPMI) (Fig 10A, 0 µM glycerol). Added glycerol had no effect on viability. As predicted, *B. hermsii* was more sensitive to MG than *B. burgdorferi*, showing significant less viability (Fig 10B) compared to *B. burgdorferi* (Fig 5B), potentially reflecting its inability to cope with carbonyl stress.

**Fig 10:**
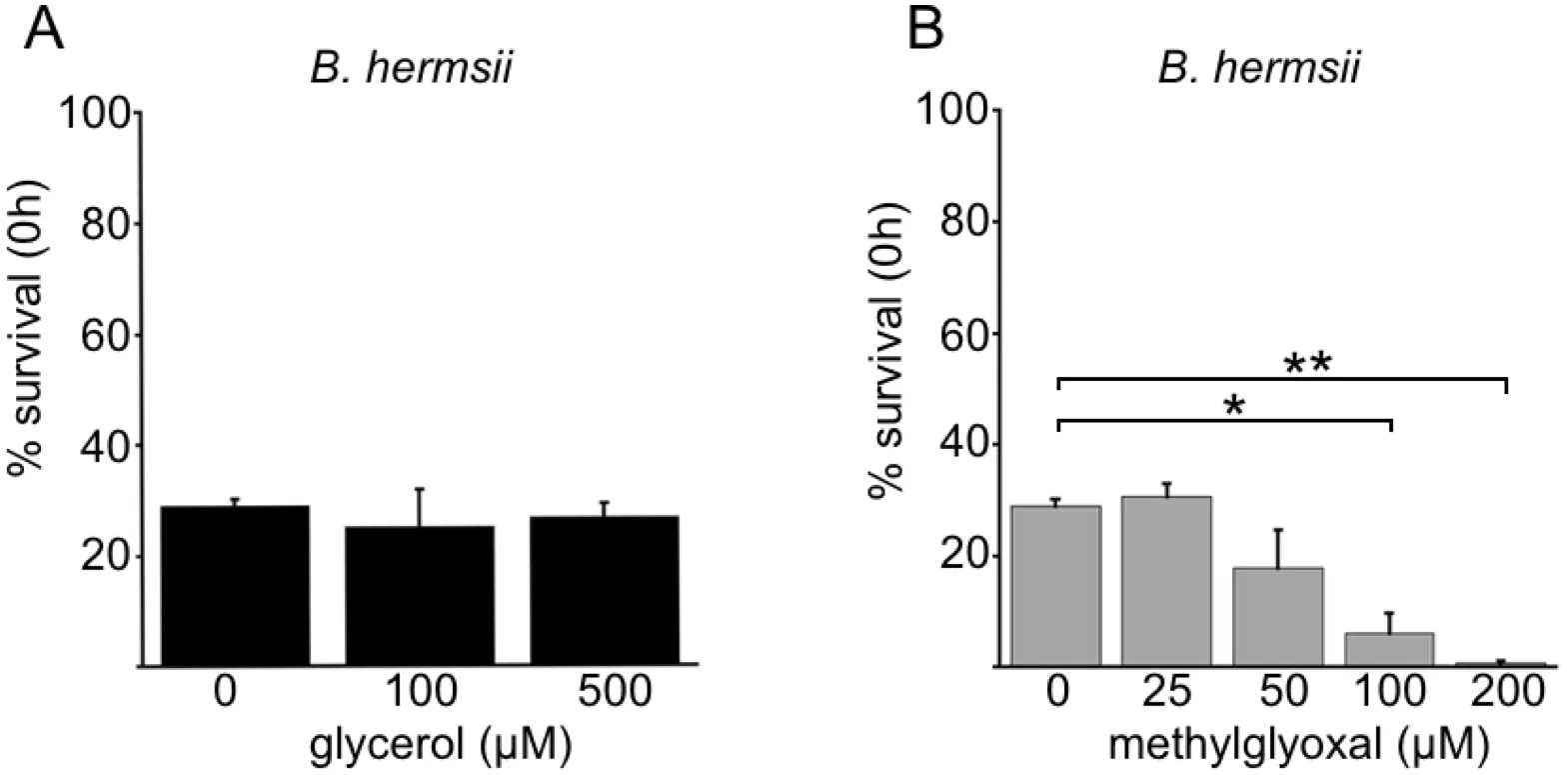
Viability of the RF spirochete *B. hermsii* in glycerol and MG. *B. hermsii* were grown in normal growth medium before shifting to RPMI medium alone or containing increasing concentrations of glycerol (A) or MG (B) for 24 h at 35°C. Cells were plated in semi-solid BSK before (0 h) and after shifting to RPMI alone (0) or increasing concentrations of glycerol or MG. Values are the mean of three independent biological replicates expressed as % survival compared to cultures before shifting to RPMI. Error bars are the SE of the mean. Asterisks represent; * *P* = 0.004 and ** P < 0.001 as determined by one-way ANOVA with a Tukey’s *post-hoc* test.

To investigate the effect of introducing the CSP into an RF *Borrelia*, we cloned the *B. burgdorferi mgsA* ORF and promoter into the endogenous lp200 plasmid of *B. hermsii* [53]. Three independent clones identified by screening for the presence of the *mgsA* gene by PCR were analyzed by immunoblot for expression of MgsA protein. All three clones (*Bh* + *mgsA*_Bb_) produced *B. burgdorferi* MgsA while wild-type *B. hermsii* was negative (Fig 11A). This, to our knowledge, represents the first case of heterologously expressing a *B. burgdorferi* gene in *B. hermsii*. To determine if the CSP is functional in *Bh* + *mgsA*_Bb_, clone 1 (Fig 11A) was grown to late log phase at 35°C and exposed to signals that induce protein glycation in *B. burgdorferi*. Wild-type *B. hermsii* and *Bh* + *mgsA*_Bb_ in normal growth medium (Fig 11B, *Bh* 0h and *Bh* + *mgsA*_Bb_ 0h) showed a single cross-reactive band, as did *B. hermsii* in 100 µM glycerol (*Bh* gly). Surprisingly, glycation was induced when *Bh* + *mgsA*_Bb_ was exposed to nutrient stress medium alone (Fig 11B, *Bh* + *mgsA*_Bb_ RPMI), indicating the CSP was functioning in *B. hermsii*. This result contrasts to the lack of glycation in *B. burgdorferi* in nutrient stress medium (Figs 2 and 3). The presence of *B. burgdorferi* glycation signals such as 100 µM or 500 µM glycerol (gly (100) and gly (500)), neopeptone (neo) or the absence of BSA (-BSA) did not increase glycation in *Bh* + *mgsA*_Bb_ over RPMI alone (Fig 11B). To examine how the CSP affects *B. hermsii* host infection and ability to relapse, we used the established animal model of relapsing fever [54]. As a control, we inserted the gentamicin resistance cassette alone in lp200, at the same locus we introduced *mgsA*_Bb_, to generate strain *Bh*+gent^R^. *Bh*+gent^R^ or *Bh*+*mgsA*_Bb_ were inoculated into four C3H-HeJ mice each and spirochetemia was followed over the course of 11 days. Starting two days after injection, blood was collected daily and spirochete load measured by qPCR and plotted as log cells per ml of blood over time with each mouse represented by a different color line (Fig 11C). The presence of the CSP in the *Bh* + *mgsA*_Bb_ strain did not affect the ability of *B. hermsii* to infect mice and produce a relapsing spirochetemia characteristic of RF. However, the peak of spirochetemia was approximately 1.5 log (32-fold) lower in mice infected with *Bh*+*mgsA*_Bb_ compared to those infected with the wild-type control (*Bh*+gent^R^) (Fig 11D). This phenotype suggests that adding the CSP and *mgsA* to *B. hermsii* suppresses the ability to replicate to high numbers in the murine host’s blood.

**Fig 11:**
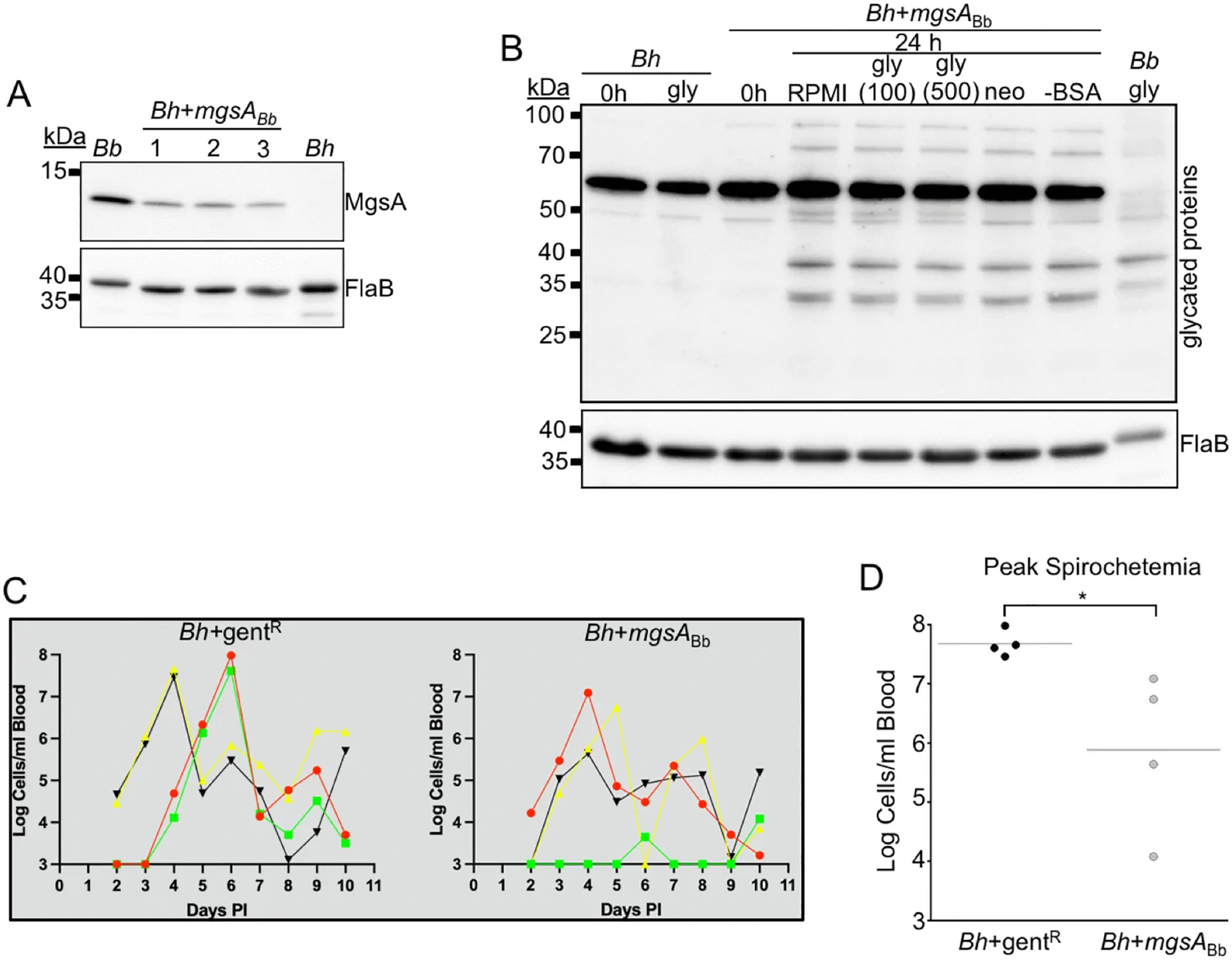
Expression of *B. burgdorferi mgsA* in the RF spirochete *B. hermsii* and effect on host infectivity. (A) *B. hermsii* was transformed with *B. burgdorferi mgsA* under control of its native *B. burgdorferi* promoter and inserted in lp200. Whole cell extracts of three independent clones, as well as wild-type *B. burgdorferi* (*Bb*) and wild-type *B. hermsii* (*Bh*), were separated by SDS-PAGE and analyzed by immunoblot with anti-MgsA antibodies to confirm MgsA expression and anti-FlaB antibodies as a loading control. **(B)** Wild-type *B. hermsii* (*Bh*) and *B. hermsii* expressing the *B. burgdorferi mgsA* with the gentamicin resistance cassette (*Bh* + *mgsA*_Bb_) were grown in normal growth medium and cell lysates were either collected (0h) or placed in nutrient stress medium (RPMI) containing 100 µM glycerol (gly 100), 500 µM glycerol (gly 500), neopeptone (neo) or BSK lacking BSA (-BSA). As a positive control for glycation, wild-type *B. burgdorferi* was incubated in RPMI containing 100 µM glycerol (*Bb* gly). After 24 h in the nutrient stress media, whole cell lysates were collected, samples were separated by SDS-PAGE and analyzed by immunoblot using antibodies against MG (glycated proteins) or FlaB. **(C)** Four mice were injected with 1 × 10^3^ cells of wild-type *B. hermsii* carrying the gentamicin resistance cassette (*Bh*+gent^R^) or four mice were injected with *B. hermsii* expressing *B. burgdorferi mgsA* (*Bh*+*mgsA*_Bb_). Starting two days post-inoculation (PI), blood was collected daily and spirochetes enumerated by qPCR. The spirochete load over time is plotted for each mouse using a different color. **(D)** The levels (log cells/ml blood) of peak spirochetemia was plotted for each mouse infected with either *Bh*+gent^R^ or *Bh*+*mgsA*_Bb_. Bars represent the mean and asterisk represents *P* = 0.04 as determined by Student’s *t*-test.

## Discussion

*B. burgdorferi* has no known or predicted effectors or secreted toxins, suggesting that many aspects of Lyme disease pathogenesis are due to the spirochetes ability to survive and/or replicate within the host. This adaptation to the host environment requires the control of bacterial metabolism in response to external signals. The regulation of bacterial metabolism, including some well-studied pathways as well as more obscure ones, to interface with disease pathogenesis represents the relatively nascent field of metabolic virulence. Here we identify the carbonyl stress pathway for the first time in the LD spirochete *B. burgdorferi*. The CSP, mediated by the metabolic intermediate MG, has been known for over 100 years, but its function remains mysterious [29–31]. Previous studies in other bacteria have implicated the CSP and MG in adaptation to environmental stress [55] and persister cell formation [56,57]. We demonstrate that the predicted *mgsA* gene encodes a functional MG synthetase that responds to external signals to glycate proteins forming AGEs. We also determine that glycerol, peptides (neopeptone) and removal of BSA from growth media are all signals that induce the CSP, while other carbohydrates such as mannose, maltose, and trehalose do not affect the CSP. This is one of the only intracellular signaling pathways in LD spirochetes where the external signal(s) inducing the pathway has been identified: the external signals inducing both the stringent response and c-di-GMP production have not yet been identified [42,43,58]. Furthermore, glycerol must be imported and metabolized to induce the CSP and not simply interact with the spirochete surface, as *glpF*, *glpK* and *glpD* null mutants are unable to induce the CSP in response to glycerol (Fig 4). Thus, there is a clear pathway through which glycerol is converted to DHAP, which serves as the substrate for MgsA to produce MG. The mechanism by which peptides (from neopeptone) intersect with DHAP metabolism or activate the CSP is unclear, as there is no defined pathway from imported peptides or amino acids to DHAP because *B. burgdorferi* lacks gluconeogenesis, the citric acid cycle, and any pathway for the *de novo* synthesis of carbohydrates [4–6]. The reason why removal of BSA from growth media induces the CSP is unknown: perhaps BSA binds to a media component or excreted product that induces the CSP; although, notably, albumin does not bind glycerol [59]. Certainly, we require a better understanding of *B. burgdorferi* metabolism and growth requirements to appreciate complex environmental adaptation strategies of this spirochete.

Previously, production of MG and the resulting AGEs have been considered cytotoxic [29,31,39,60,61], but we demonstrate here that addition of 100 µM MG, while resulting in protein glycation, did not decrease *B. burgdorferi* survival (Figs 2 and 5). Curiously, the *mgsA* mutant was completely resistant to the cytotoxicity of glycerol (Fig 5C) suggesting that MgsA, and, presumably, the carbonyl stress pathway, but not MG, are responsible for the lack of viability in glycerol. Thus, our results suggest that a factor related to MG production causes glycerol-induced cytotoxicity, such as increased inorganic phosphate levels during MG synthesis or a metabolic intermediate associated with MG metabolism. Furthermore, LD spirochetes, which harbor an *mgsA* gene and, thus, a CSP, were more resistant to added MG than RF spirochetes (*B. hermsii* survival decreased in 100 µM MG, Fig 10), which lack a predicted *mgsA* ortholog, suggesting a potential genetic link to MG tolerance. The LD spirochetes may experience increased MG levels during persistence in their tick vectors, and the absence of *mgsA* in RF spirochetes may reflect the different species of ticks that vector these bacteria or the local environment during persistence in the tick: LD spirochetes reside in the midgut of hard ticks and RF spirochetes are primarily located in the salivary glands of soft ticks, which is a consequence of the different kinetics of feeding by the two groups of ticks [2,62].

We have identified several factors and gene products, aside from the *glp* operon, that regulate the CSP in *B. burgdorferi*. Intracellular second messenger systems, such as the stringent response mediated by (p)ppGpp and the cyclic-di-GMP network, integrate external nutrient-related signals to adapt *B. burgdorferi* to its environment [3,42,43]. These systems modulate a variety of intracellular processes including transcription, translation and metabolism. We found that the stringent response affected the CSP when induced by glycerol, as the *rel*_Bbu_ mutant had lower levels of glycation compared to the wild-type and complemented strains. This is likely because expression of the *glp* operon, containing the genes encoding the proteins for glycerol transport and metabolism, is Rel_Bbu_-dependent, thus less glycerol would enter the cell and be converted to DHAP [15,16]. The c-di-GMP-binding protein PlzA likely affects the CSP similarly, with reduced glycation seen in a *plzA* null mutant. Curiously, the *rrp1* null mutant, which does not produce c-di-GMP, has the opposite phenotype: glycation is increased in the mutant even though *glp* operon expression is lower in this strain and less glycerol is expected to enter the cell. These findings suggest holo-PlzA represses glycation (higher glycation in *rrp1* null mutant that does not produce c-di-GMP, Fig 7C) while apo-PlzA increases glycation (lower glycation in *plzA* null mutant, Fig 7D); holo-PlzA and apo-PlzA are known to have different activities and physiological functions [28,48,63]. Likely, other internal metabolic or regulatory factors also have a role regulating the CSP and glycation.

Glycerol metabolism has been linked to bacterial pathogenesis in *Pseudomonas aeruginosa* [64], *Vibrio splendidus* [65] and *E. coli* persisters [66]. The CSP and *mgsA* are not essential for vertebrate infection but have a modest effect at a low *B. burgdorferi* inoculum (Table 2). Persistence in the tick in the animal model of Lyme disease is independent of the CSP, but glycation may play a role in the host response to *B. burgdorferi* infection. Glycation of *B. burgdorferi* proteins may modulate the host immune response during colonization of ticks and/or vertebrates as AGEs have been implicated in numerous chronic diseases such as cancer, heart disease, diabetes, and neurological impairments, albeit this hypothesis remains speculative [36–38].

RF spirochetes lack a predicted *mgsA* ortholog and thus a CSP (Table 1) [51,52]. *B hermsii* responds differently than *B. burgdorferi* to nutrient stress (RPMI) and glycerol: *B. hermsii* is killed more readily in response to nutrient stress, but glycerol does not exacerbate this effect (Fig 10). Additionally, when *mgsA*_Bb_ was expressed in *B. hermsii*, the CSP and glycation were activated by nutrient stress alone and this activation was not increased by adding glycerol, as in *B. burgdorferi*. The contrasting responses of RF and LD spirochetes to nutrient availability may reflect the nutrients present in their different environments: either in the tick, where RF spirochetes reside in salivary glands and LD spirochetes persist in the midgut, or in the host, where RF spirochetes replicate in the blood and LD spirochetes disseminate throughout the host. In fact, RF spirochetes are thought to be able to replicate to high numbers in blood because they possess a more complete glycerol metabolic pathway that includes glycerophosphodiester phosphodiesterase (*glpQ*) and glycerol-3-phosphate transporter (*glpT*) while LD spirochetes lack these homologs (Table 1) [67]. Another possibility is that GlpQ and GlpT are important for glycerol metabolism and survival in ovaries, as RF spirochetes can transmit transovarially, unlike LD spirochetes [2,62]. Interestingly, *B. mayonii*, an LD spirochete that can replicate to high numbers in the blood, has an *mgsA* gene, but expresses low levels of MgsA protein compared to *B. burgdorferi* and does not induce the CSP and glycation in response to glycerol similar to the RF spirochetes (Fig 9B and C). During murine infection, *B. hermsii* expressing the CSP from *B. burgdorferi* (*mgsA*_Bb_) were unable to reach the same peak of spirochetemia as wild type, suggesting that the CSP may curtail robust replication, at least in host blood (Fig 11). Not all LD spirochete species induce the CSP in response to glycerol: *B. afzelii* and *B. bissettiae* fail to glycate proteins and *B. andersonii* showed persistent glycation (S2 Fig). While these species group with the *B. burgdorferi*, they present different disease pathogenesis, as *B. bissettiae* does not typically infect humans, *B. afzelii* infection is primarily in the skin, and *B. andersonii* is also not associated with human disease. This study represents the first expression of an LD spirochete gene in an RF spirochete, to our knowledge. The heterologous expression of *mgsA* provides evidence that glycerol metabolism is important for spirochetemia and suggests the raison d’être for RF spirochetes having lost the CSP from their genome. Taken together, these results suggest that an absence of the CSP, in RF spirochetes, or a subdued CSP that does not respond to glycerol, as in *B. mayonii*, may be an evolutionary adaption to replicate to high numbers in the blood of the host, which facilitates acquisition by feeding ticks.

## Materials and methods

**Ethics Statement.** All animal protocols were approved by the University of Montana Institutional Animal Care and Use committee (*B. burgdorferi* experiments) or the Rocky Mountain Laboratories, National Institute of Infectious Diseases, National Institutes of Health Animal Care and Use Committee (*B. hermsii* experiments, protocol number 2018-061). All experiments followed the *Guidelines for the Care and Use of Laboratory Animals* from the National Institutes of Health. The University of Montana and the Rocky Mountain Laboratories are both accredited by the International Association for Assessment and Accreditation of Laboratory Animal Care (AAALAC).

### *Borrelia* strains and growth conditions

Low-passage *B. burgdorferi* B31-5A4 [68], 297 (BbAH130) [69] *B. afzelii* PKo [70], *B. bissettiae* DN127 [71]*, B. andersonii* 21038 [72], *rel*_Bbu_ [16], *glpD* [18], *dksA* [27], *rrp1* [14], *plzA* [47], *mgsA* mutant and complemented strains, and *B. mayonii* MN14-1420 [49] were grown and maintained in gelatin-free Barbour-Stoenner-Kelly II (BSK) liquid medium, pH 7.6, containing 6% rabbit serum (RS) (Pel-Freez Biologicals) [73]. Cultures were grown at 35°C to 5–9 × 10^7^ cells ml^−1^ for experiments, unless noted otherwise. Cell counting was done using a Petroff-Hausser cell counting chamber [74]. *B. hermsii* DAH [51] and *B. parkeri* were grown in BSK + 12% RS at 35°C. All strain sources are listed in S2 Table.

### *B. burgdorferi* strain construction

The parental strain B31-5A4, referred to as wild type, or B31-5A4 with the gentamicin resistance cassette in *bbe02* on lp25 (B31-5A4/*bbe02*-gent^R^) were used to construct all mutant strains. *E. coli* TOP10F′ grown in lysogeny broth were used for cloning. The *mgsA* null mutant was constructed as previously described [74] by amplifying the regions upstream and downstream of the *mgsA* gene by PCR with B31-5A4 genomic template DNA using the primers mgsA_U435F with mgsA_D424R+AatII + AgeI and mgsA_380F + AatII with mgsA_D1377 + AgeI (S1 Table), respectively. PCR products were separated by gel electrophoresis, gel purified, poly-A tailed and cloned into pCR2.1-TOPO (Invitrogen) before DNA sequencing to verify fidelity. Plasmids containing the two DNA segments were digested using AgeI and AatII restriction enzymes and the resulting pieces separated by agarose gel electrophoresis, gel-extracted and ligated together at the engineered AatII restriction site. The streptomycin/spectinomycin resistance cassette with the *flgB* promoter from *B. burgdorferi* [75] and *trpL* terminator from *Bacillus subtilis* (*flgBp*-*aadA*-*trpLt*) containing flanking AatII sites was placed between the upstream and downstream DNA segments. This *mgsA*-knockout plasmid was purified and linearized using the restriction enzyme AhdI before electroporating competent B31-5A4 cells. One day after transformation, cells were vortexed, plated in liquid BSK + RS containing 50 μg ml^−1^ streptomycin for selection in 96-well plates. Wells containing growing *B. burgdorferi* were screened for replacement of the *mgsA* gene with the streptomycin resistance cassette using the primers mgsA_U108F and mgsA_D449R (S1 Table). The *mgsA cis*-complemented strain was generated by amplifying the *mgsA* ORF and promoter by PCR using the primers mgsA_U435F and mgsA_D424R+AatII + AgeI to yield the upstream segment. This upstream segment (containing the *mgsA* ORF) and the same downstream segment used to make the knockout construct described above were digested with AatII and AgeI. The same strategy used to generate the *mgsA* knockout construct was used for the *cis*-complement construct with the exception that the gentamicin resistance cassette (P*flgB*-*aacC1*) was placed downstream of the *mgsA* gene. See Fig 3A for a graphic depiction. Competent *mgsA* knockout cells were transformed with the linearized *mgsA cis-*complement construct and mutants selected for in 40 μg ml^−1^ gentamicin. Mutants were screened as described for the *mgsA* mutant.

### Construction of the *glpF* mutant

The *glpF* null mutant was constructed by first amplifying the regions upstream and downstream of the *glpF* gene by PCR with B31-5A4 genomic template DNA using the primers glpF_U1203F with glpF_3R + Nd + Aa + Ag and glpF_754F + AatII with glpF_D1720R+AgeI (S1 Table), respectively. Upstream and downstream DNA segments were combined as described above. The kanamycin resistance cassette, lacking a promoter and terminator, containing 5′ NdeI and 3′ AatII restriction sites was placed between the upstream and downstream DNA segments by digestion and ligation of the cassette and *glpF* segments. This *glpF*-knockout plasmid was transformed into *E. coli*, purified and linearized using the restriction enzyme AhdI before electroporating competent B31-5A/*bbe02*-gent^R^ cells. 24 h after transformation, cells were vortexed, plated in liquid BSK + RS containing 40 μg ml^−1^ gentamicin and 800 μg ml^−1^ kanamycin for selection in 96-well plates. Wells containing growing *B. burgdorferi* were screened for replacement of the *glpF* gene with the kanamycin resistance cassette using the primers glpF_D1720R+AgeI and kan_88F (S1 Table). The *glpF cis*-complemented strain was generated by amplifying a region upstream from the *glpF* ORF by PCR using the primers glpF_1203F + AgeI and glpF_U163R+AatII to yield the upstream segment. The primers glpF_U359F+AatII + AgeI and glpF_D1720R were used to construct the DNA containing the *glpF* ORF and downstream segment, and both pieces cloned and sequenced verified as above. The two pieces were digested with AatII and AgeI and the upstream DNA ligated to the *glpF* ORF and downstream segment. The streptomycin/spectinomycin resistance cassette containing the *flgB* promoter and *trpL* terminator flanked by AatII restriction sites was placed between the upstream and downstream DNA segments, resulting in the cassette being upstream of the *glp* operon promoter, by digestion and ligation of the cassette and *glpF* segments. This *glpF*-complement plasmid was transformed into *E. coli*, purified and linearized using the restriction enzyme AhdI before electroporating competent B31-5A/*bbe02*-gent^R^ ∆*glpF* mutant cells. Transformants were selected for as described above using 40 μg ml^−1^ gentamicin and 50 μg ml^−1^ streptomycin for selection in 96-well plates. *glpF*-complemented clones were confirmed by the presence of the *glpF* gene by PCR using the primers glpF_1F + SacI and glpF_282R (S1 Table).

### Construction of the *glpK* mutant

The *glpK* null mutant was constructed by first amplifying the regions upstream and downstream of the *glpK* gene by PCR with B31-5A4 genomic template DNA using the primers glpK_U1213F with glpK_3R + Nd + Aa + Ag and glpK_1506F + AatII with glpK_D2387R+AgeI (S1 Table), respectively. Upstream and downstream DNA segments were combined as described above. The kanamycin resistance cassette, lacking a promoter and terminator, containing 5′ NdeI and 3′ AatII restriction sites was placed between the upstream and downstream DNA segments by digestion and ligation of the cassette and *glpK* segments. This *glpK*-knockout plasmid was transformed into *E. coli*, purified and linearized using the restriction enzyme AhdI before electroporating competent B31-5A/*bbe02*-gent^R^ cells. 24 h after transformation, cells were vortexed, plated in liquid BSK + RS containing 40 μg ml^−1^ gentamicin and 800 μg ml^−1^ kanamycin for selection in 96-well plates. Wells containing growing *B. burgdorferi* were screened for replacement of the *glpK* gene with the kanamycin resistance cassette using the primers glpK_D2387R+AgeI and kan_88F (S1 Table). To generate the *glpK* complemented strain, a streptomycin/spectinomycin resistance cassette, lacking a promoter and terminator, was placed downstream of the *glpK* gene by replacing the vestigial ORF *bb0242*. The upstream DNA segment (containing the *glpK* ORF) was made by PCR using the primers glpK_U526F and bb242_3R + Nd + Aa + Ag and the downstream piece made with primers bb242_278F + AatII and bb242_D1161R+AgeI. The two pieces were digested with AatII and AgeI and the upstream DNA ligated to the *glpK* ORF and downstream segment. The streptomycin/spectinomycin resistance cassette lacking a promoter and terminator flanked by NdeI and AatII restriction sites was placed between the upstream and downstream DNA segments, by digestion and ligation of the cassette and *glpK* segments. This *glpK*-complement plasmid was transformed into *E. coli*, purified and linearized using the restriction enzyme AhdI before electroporating competent B31-5A/*bbe02*-gent^R^ ∆*glpK* mutant cells. Transformants were selected for as described above using 40 μg ml^−1^ gentamicin and 50 μg ml^−1^ streptomycin for selection in 96-well plates. *glpK*-complemented clones were confirmed by the presence of the *glpK* gene by PCR using the primers glpK_630F and glpK_913R + T7.

The *glpD* null mutant and complemented strains were previously described [18]. All genetically modified strains were screened by multiplex PCR to ensure retention of *B. burgdorferi* linear and circular plasmids [76].

### *B. hermsii* strain construction

The heterologous expression of the *B. burgdorferi mgsA* gene in *B. hermsii* was done by inserting the cloned *B. burgdorferi mgsA* gene, containing the predicted promoter, into the *B. hermsii* linear plasmid 200 (lp200) at the same site that was previously used to insert *gfp* [53]. The primers VC_1 + VC_2 + XmaI + AatII and VC_7 + XmaI + VC_8 + AatII were used to amplify two adjacent regions of a noncoding region of lp200 using *B. hermsii* DAH genomic DNA as a template. The VC_1/VC_2 segment was cloned into pCR-XL-2-TOPO. The two segments of DNA were digested with XmaI and AatII and ligated together using the strategy described above for *B. burgdorferi* to yield pTA-lp200-part. Next, we cloned the *B. burgdorferi mgsA* gene and promoter and a gentamicin selectable marker, which together were inserted between the two segments of lp200 described above. The *mgsA*_Bb_ gene and its promoter were amplified by PCR with primers mgsA_U435F+XmaI and mgsA_D424R+AatII + AgeI using *B. burgdorferi* wild-type genomic DNA as template. The gentamicin resistance cassette for *B. hermsii* was amplified by PCR using the primers BhflaBp_F + AatII and aacC1_R + XmaI + AgeI using the plasmid pTABhFlaB-Gent as template [77]. After cloning into pCR-TOPO 2.1, DNA segments were digested with AatII and AgeI and ligated as described above to yield pTA-mgsABb + BhFlaB-Gent. This plasmid and the plasmid pTA-lp200-part containing part of lp200 were digested with XmaI and *mgsA*_Bb_+ BhFlaB-Gent inserted into pTA-lp200-part to give pTA-lp200-part-*mgsA*_Bb_- BhFlaB-Gent. As a control, the BhFlaB-Gent cassette was inserted into pTA-lp200-part to give pTA-lp200-part-BhFlaB-Gent. The pTA-lp200-part-*mgsA*_Bb_- BhFlaB-Gent or pTA-lp200-part-BhFlaB-Gent constructs were linearized by digestion with AhdI and purified linear plasmids used to transform *B. hermsii* and select for clonal mutant strains in 40 μg ml^−1^ gentamicin as previously described [77]. Positive colonies were screened by PCR for the presence of the gentamicin cassette using the primers aacC1_654F and aacC1R+AatII. Production of MgsA_Bb_ in the transformed *B. hermsii* clones was confirmed by immunoblot using anti-MgsA antibodies (see Immunoblot analyses section below).

### Viability under nutrient stress

*B. burgdorferi* and *B. hermsii* strains were grown to 4–9 × 10^7^ cells ml^−1^, a portion diluted in BSK, and plated in semi-solid BSK (0 h time point). The remaining cultures were collected by centrifugation at 8,000 x *g* for 10 min at 4°C. Cells were resuspended in RPMI 1640 without L-glutamine (Corning) either without or with the indicated concentrations of glycerol (Fisher Scientific) or methylglyoxal (Sigma) and incubated at 35°C for the times indicated. Viability was assayed by plating in semi-solid BSK and colony enumeration as previously described for *B. burgdorferi* [16] and *B. hermsii* [78]. Approximately two weeks after plating colonies were enumerated and percent survival was calculated by (# of colonies following treatment / # of colonies at 0 h) * 100. Values are the means of at least three independent biological replicates.

### Immunoblot analyses

Whole cell *B. burgdorferi* extracts were analyzed by SDS-PAGE using Novex 4–20% Tris-Glycine polyacrylamide gels (Invitrogen). Proteins transferred to PVDF Immobilon membranes (Millipore), blocked overnight at 4°C in blocking buffer consisting of dPBS + 0.5% Tween-20 + 4% dried milk + 1% goat serum and incubated with antibodies diluted in blocking buffer: mouse anti-MG (1:2500, Cell Biolabs), rat anti-MgsA (1:1000), mouse anti-FlaB (1:100, gift from Tom Schwan). Purified MgsA protein was provided by the Seattle Structural Genomics Center for Infectious Disease (www.SSGCID.org). Polyclonal rat anti-MgsA antibodies were produced by General Bioscience Corporation using purified MgsA protein from SSGCID. Primary antibodies were detected with goat anti-rat or goat anti-mouse HRP-linked antibodies (1:15,000, Bio-Rad Laboratories). Antigen-antibody complexes were detected using chemiluminescence (Amersham ECL Prime, GE Healthcare) and visualized using an LAS-3000 Intelligent Dark Box (Fujifilm Medical Systems USA). Images are representative of immunoblots that were repeated at least twice.

### *B. burgdorferi* mouse infectivity by needle inoculation and tick transmission

C3H-HeJ mice (Charles River Laboratories) were injected intradermally with 1 × 10^3^ to 1 × 10^4^ *B. burgdorferi* cells grown at 35°C to cell densities of 5–9 × 10^7^ cells ml^−1^. To examine the infectivity of *B. burgdorferi* strains, ear biopsies were collected three-weeks post inoculation and tissues cultured in in BSK II containing 50 µg ml^−1^ rifampicin, 20 µg ml^−1^ phosphomycin and 2.5 µg ml^−1^ amphotericin B. Successful infection was determined by spirochete outgrowth using darkfield microscopy within 14 days. Five weeks post-inoculation, mice were sacrificed and ear, ankle and bladder tissues were collected, cultured and examined for spirochetes.

For tick acquisition and transmission studies, approximately 100 naive *Ixodes scapularis* larvae (National Tick Research and Education Resource, Oklahoma State University) were allowed to feed to repletion on infected mice. Collected fed larvae were maintained in a 98% humidified chamber until molting into nymphs (about 10 weeks) and after collection of fed nymphs. To examine transmission, three larvae were allowed to feed to repletion on three mice. Murine transmission was monitored by culturing ear, ankle and bladder tissues as described above. Persistence of *B. burgdorferi* in fed larvae, unfed nymphs and fed nymphs was followed by qPCR as described below.

### Quantification of *B. burgdorferi* in ticks

Spirochete burdens in ticks were assessed in fed larvae (one week, in groups of five), unfed nymphs (in groups of five) and fed nymphs (one-week post-feeding to repletion). DNA was extracted from ticks using the DNeasy Blood/Tissue kit (Qiagen) and the number of *B. burgdorferi* genomes in each tick determined by TaqMan qPCR with primers flaB_425F and flaB_542R and the flaB probe to the *flaB* gene (S1 Table) where 1 copy of *flaB* = one genome as previously described [16].

### *B. hermsii* infection of mice and quantification in murine blood

Mice were infected with *B. hermsii* DAH or DAH + *mgsA*_Bb_ and spirochete levels in the blood quantified as previously described [78]. Briefly, female RML (Rocky Mountain Laboratories) mice were needle inoculated intraperitoneally with 1 × 10^3^ *B. hermsii* cells. At the days indicated post-inoculation, three samples of five µl of blood were collected by tail vein bleed and stored at -80°C in SideStep Lysis and Stabilization buffer (Agilent Technologies). The levels of *B. hermsii* in the samples was quantified at the same time by TaqMan qPCR using Stratagene Brilliant II qPCR Master Mix (Agilent Technologies) and primers and probe to the *B. hermsii flaB* gene (S1 Table). The number of spirochetes was calculated using a standard curve of a known number *of B. hermsii* in Stabilization buffer in a series of tenfold dilutions [79].

## Supporting information

S1 Table Oligonucleotides used in this study. (DOCX)

S2 Table Strain sources. (DOCX)

S1 Fig Raw images of immunoblots and gels. (PDF)

S2 Fig Protein glycation in *Borrelia* (*Borreliella*) spp. *B. afzelii, B. bissettiae and B. andersonii* were grown to ~7 × 10^7^ cells ml^−1^ in BSK + RS before collection by centrifugation (BSK + RS) or resuspended in 100 µM glycerol in RPMI for 24 h at 35°C. Equal amounts of whole cell lysates were separated by SDS-PAGE and visualized by immunoblot analyses with anti-MG antibodies to detect protein glycation (upper panel) or anti-MgsA antibodies (middle panel), and anti-FlaB antibodies as a loading control.
(TIF)
