# Supplementary material for: Metabolic regulation of the carbonyl stress pathway and methylglyoxal in Lyme disease and relapsing fever spirochetes
Source: PLoS Pathog. 2026 Sep 29;22(9):e1014657. doi: 10.1371/journal.ppat.1014657 (PMC13641704; doi:10.1371/journal.ppat.1014657)

Fig. 2A: 1. WT 0h 2. - 3. 10 gly 4. 30 gly 5. 100 gly 6. 10 MG 7. 30 MG 8. 100 MG

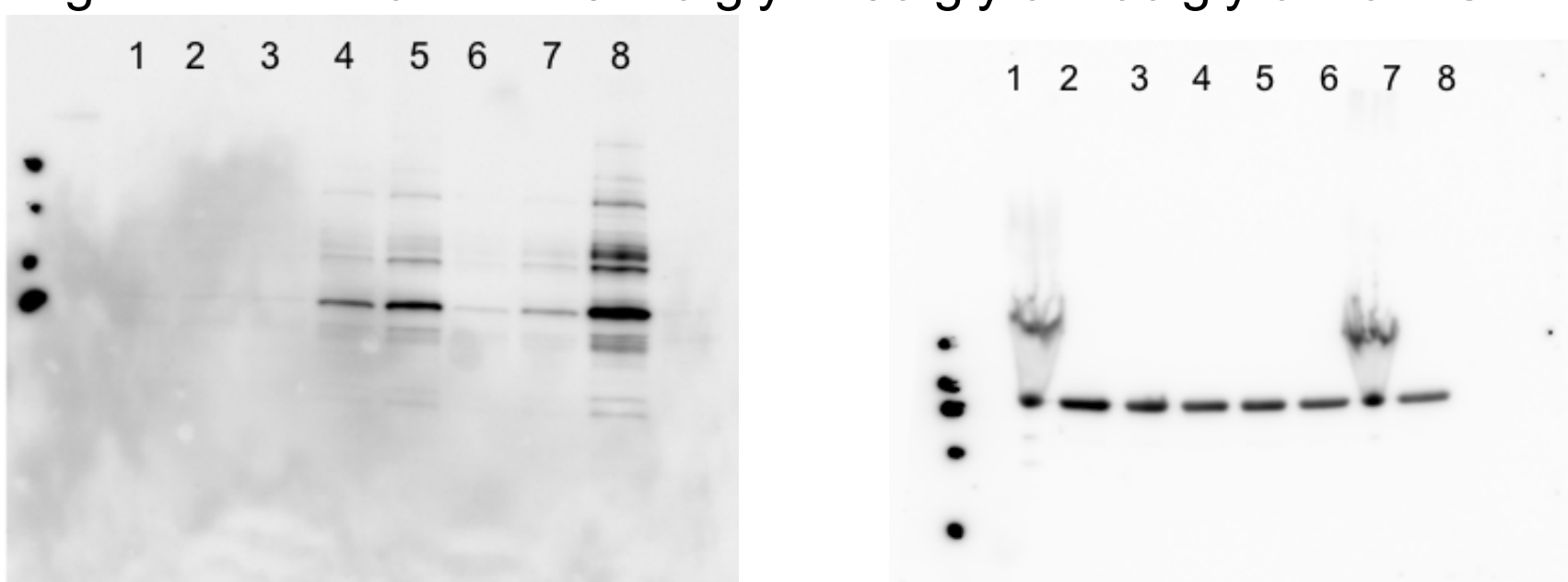

Fig 2B: 1. 0h 2. RPMI 3. RPMI+neopeptone 4. BSK-BSA-RS

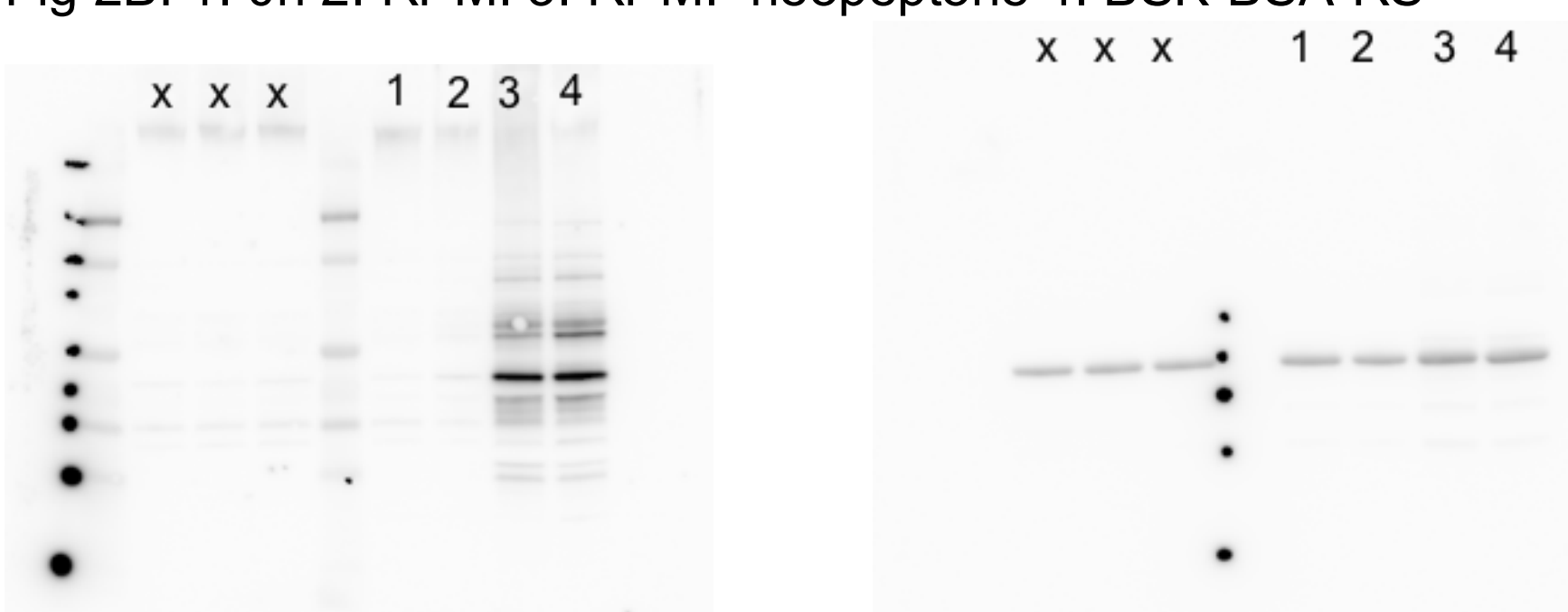

Fig 2C: 1. 0h 2. – 3. +BSA 4. Glycerol +RS 5. Glycerol +BSA+RS 6. Mannose 7. Maltose 8. Trehalose 9. GlcNAc

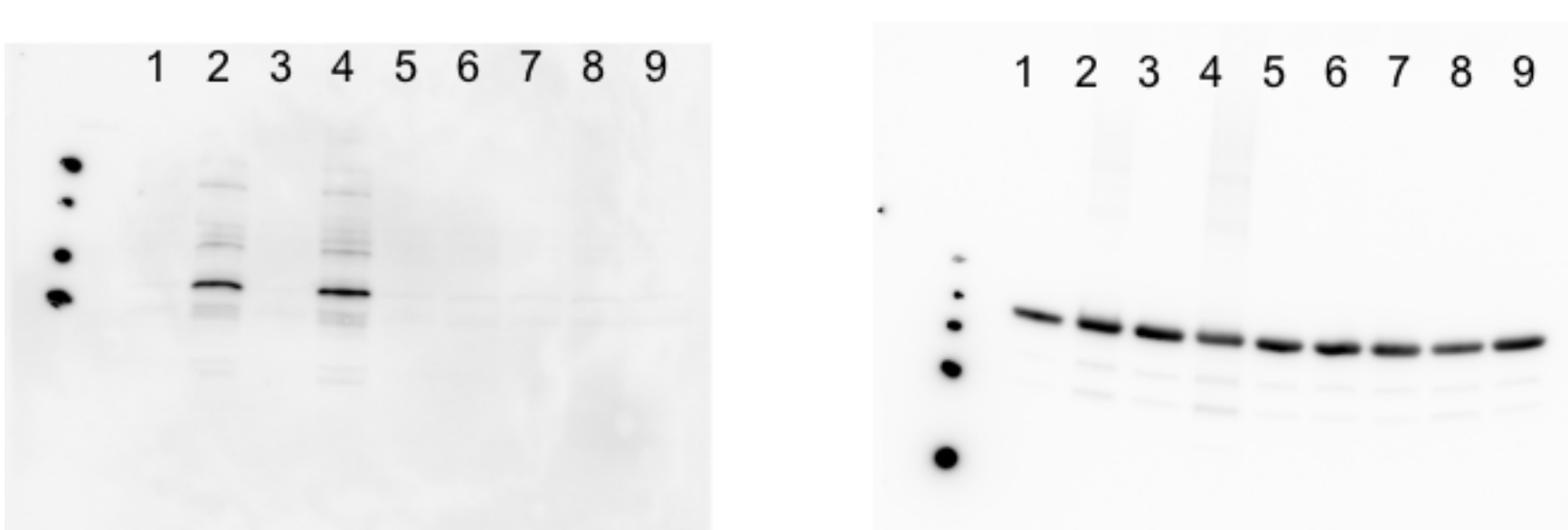

Fig 3B: 1. WT 2. Δ*mgsA* 3. *mgsA*+

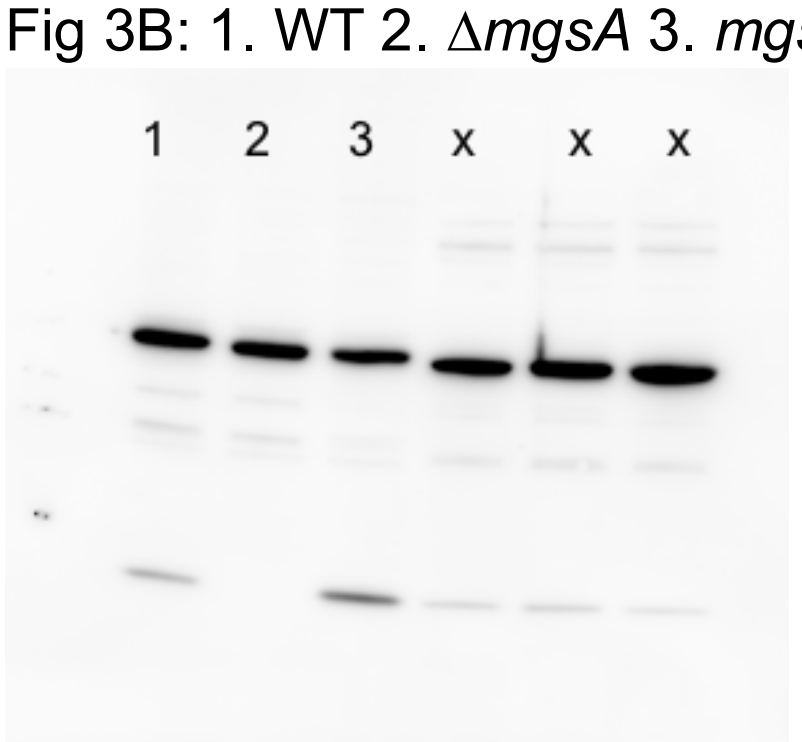

Fig 3C: 1. WT RPMI 2. Δ*mgsA* RPMI 3. *mgsA*+ RPMI 4. WT glycerol 5. Δ*mgsA* glycerol 6. *mgsA*+ glycerol 7. WT 100 MG 8. Δ*mgsA* 100 MG 9. *mgsA*+ 100 MG

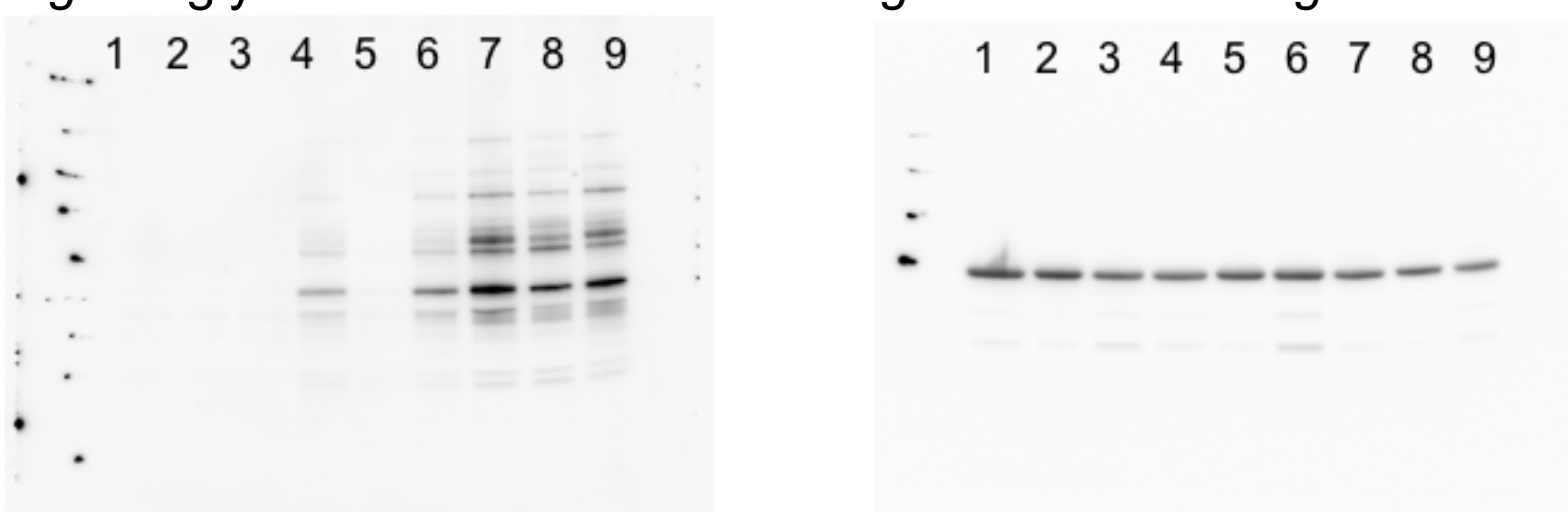

Fig 3D: 1. WT RPMI+neopeptone 2. Δ*mgsA* RPMI+neopeptone 3. *mgsA*+ RPMI+neopepetone 4. WT BSK-RS-BSA 5. Δ*mgsA* BSK-RS-BSA 6. *mgsA*+ BSK-RS-BSA

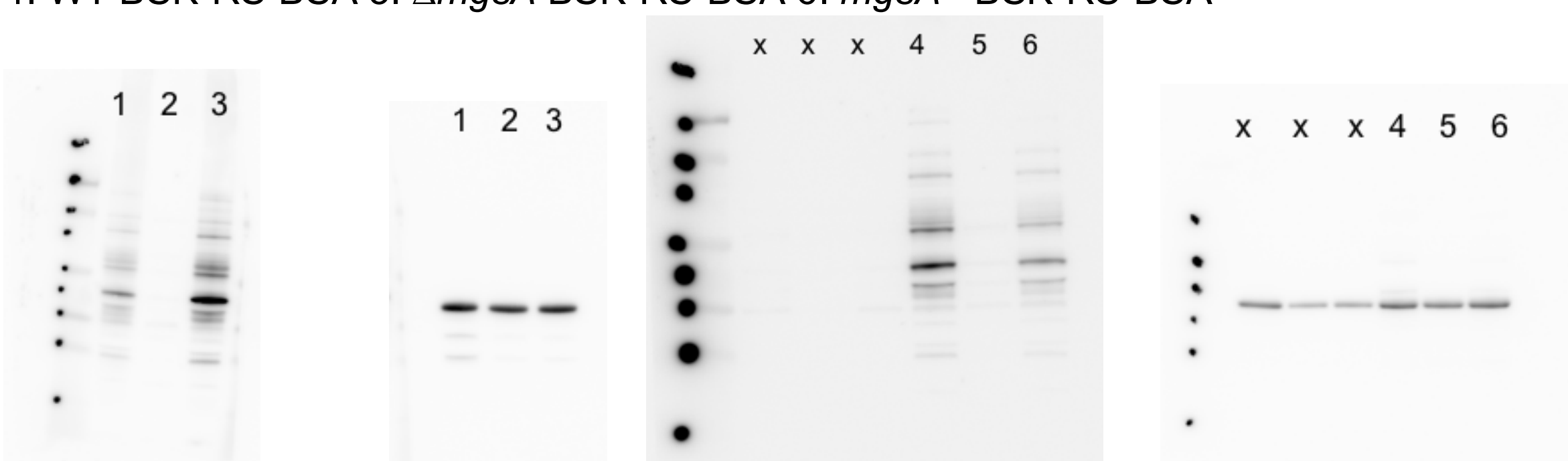

Fig 4: 1. WT glycerol 2. Δ*glpF* glycerol 3. *glpF*+ glycerol 4. Δ*glpK* glycerol 5. *glpK*+ glycerol 6. WT glycerol 7. Δ*glpD* glycerol 8. *glpD*+ glycerol

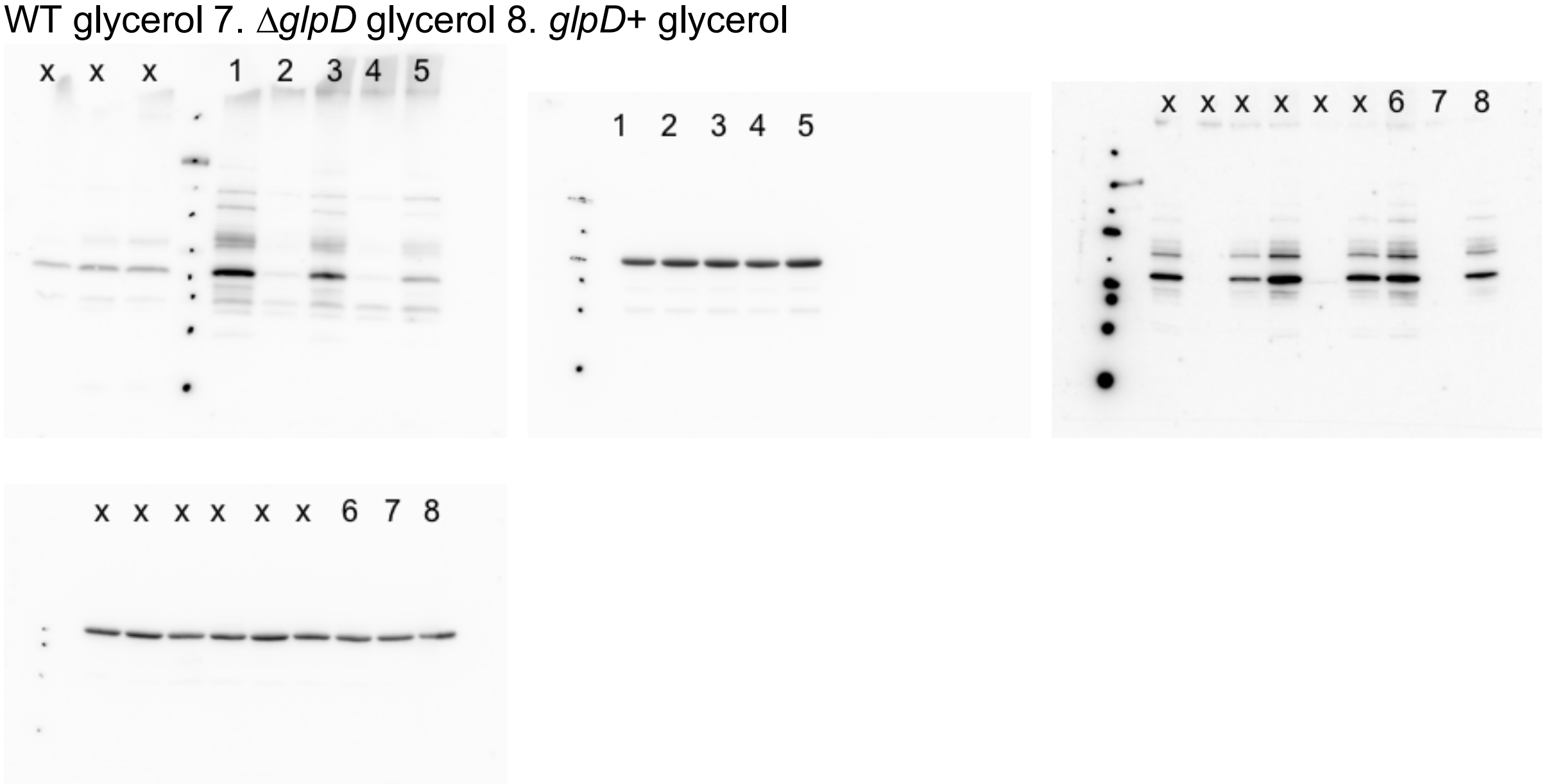

Fig 6A: 1. Mid-log 2. Late log 3. Stationary 4. Late stationary

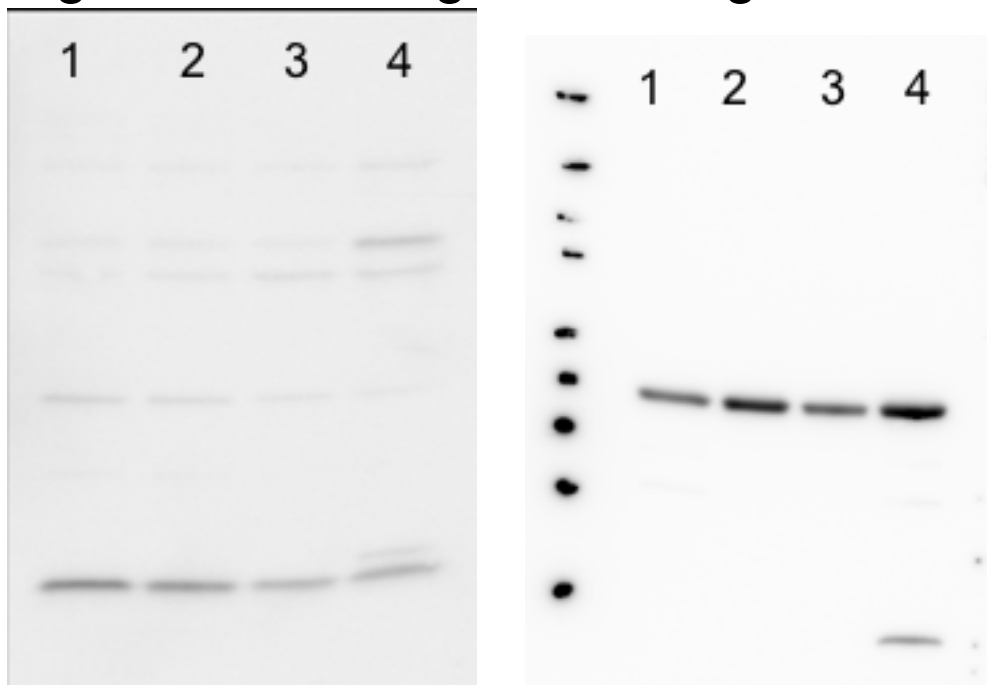

Fig 6B: 1. Mid-log 0h 2. Mid-log -24h 3. Mid-log gly 24h 4. Late log 0h 5. Late log -24h 6. Late log gly 24h 7. Late stat 0h 8. Late stat -24h 9. Late stat gly 24h

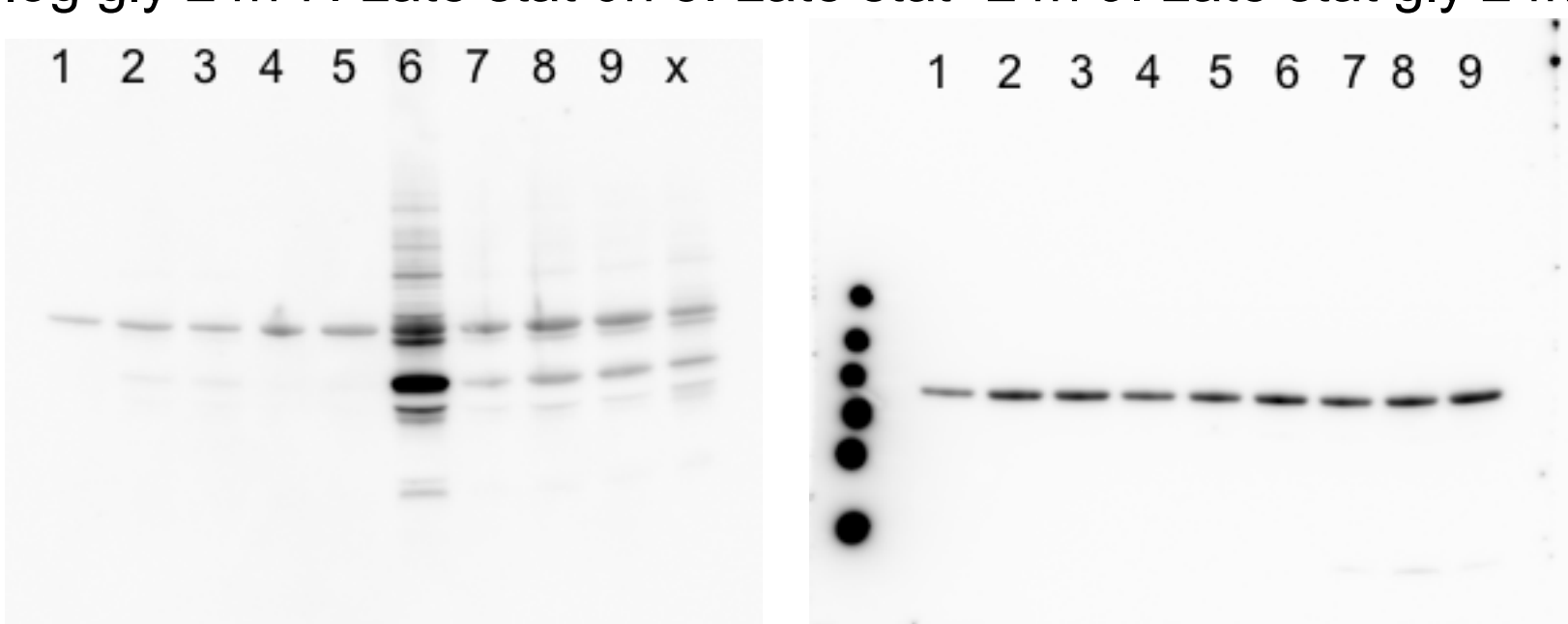

Fig 7A: 1. WT BSK+RS 2. Δ*rel* BSK+RS 3. *rel*+ BSK+RS 4. WT glycerol 5. Δ*rel* glycerol 6. *rel*+ glycerol 7. WT neopeptone 8. Δ*rel* neopeptone 9. *rel*+ neopeptone

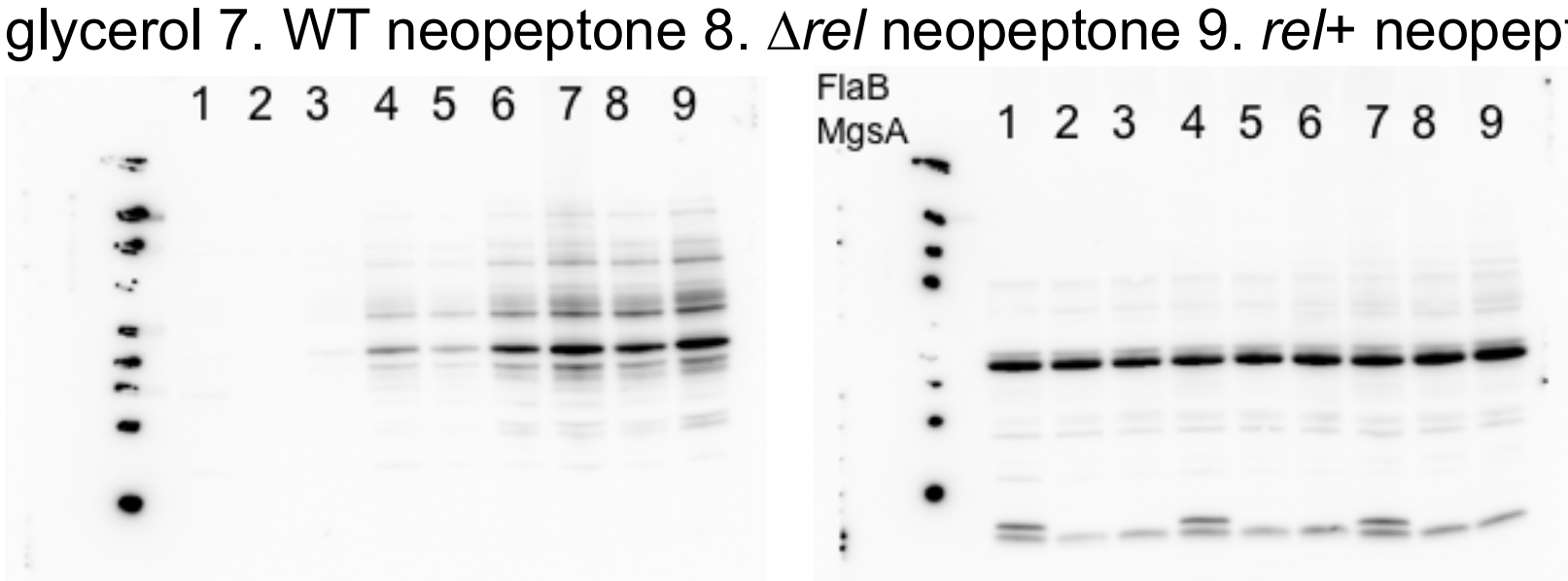

Fig 7B: 1. WT BSK+RS 2. Δ*dksA* BSK+RS 3. *dksA*+ BSK+RS 4. WT glycerol 5. Δ*dksA* glycerol 6. *dksA*+ glycerol 7. WT neopeptone 8. Δ*dksA* neopeptone 9. *dksA*+ neopeptone

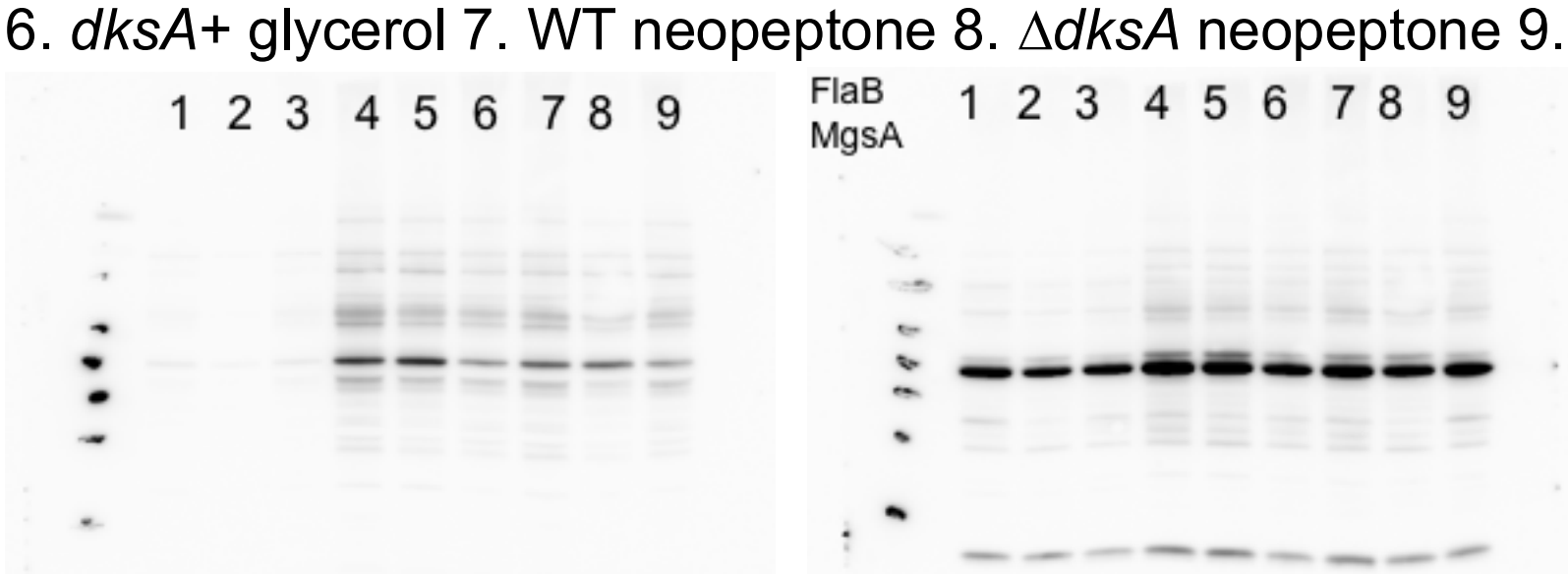

Fig 7C: 1. WT BSK+RS 2. Δ*rrp1* BSK+RS 3. *rrp1*+ BSK+RS 4. WT glycerol 5. Δ*rrp1* glycerol 6. *rrp1*+ glycerol 7. WT neopeptone 8. Δ*rrp1* neopeptone 9. *rrp1*+ neopeptone

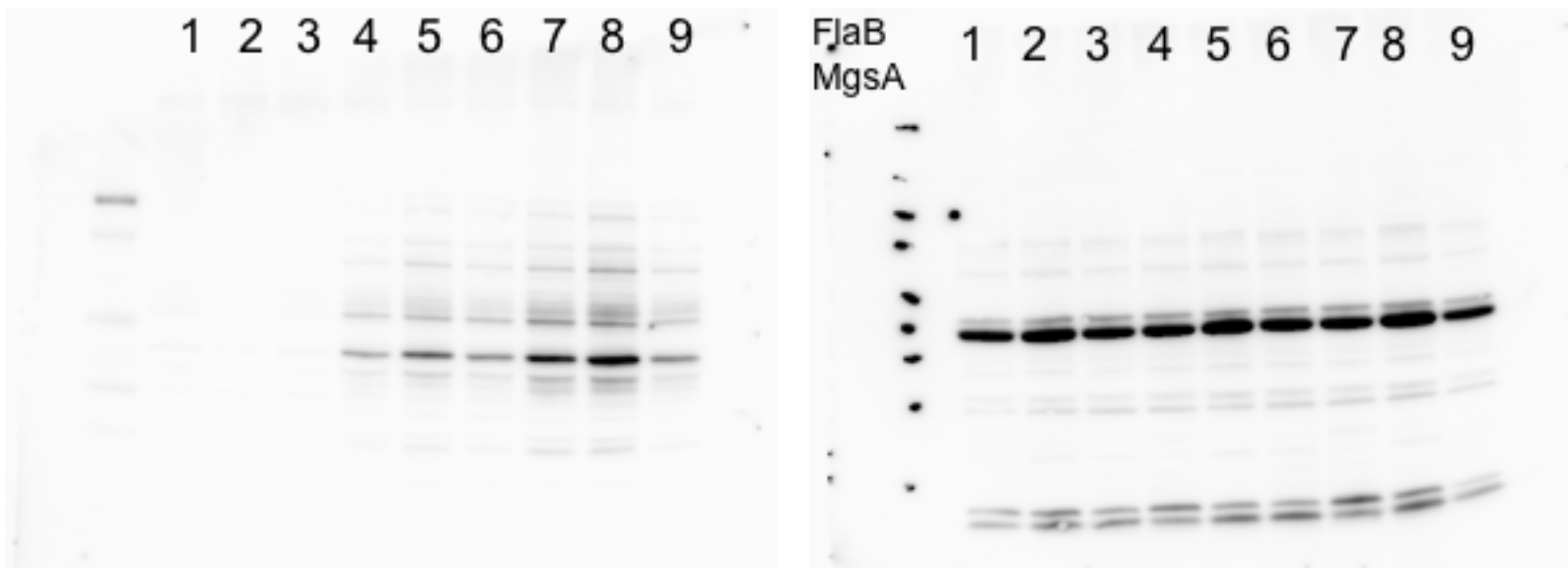

Fig 7D: 1. WT BSK+RS 2. Δ*plzA* BSK+RS 3. *plzA*+ BSK+RS 4. WT glycerol 5. Δ *plzA* glycerol 6. plzA+ glycerol 7. WT neopeptone 8. Δ *plzA* neopeptone 9. *plzA*+ neopeptone

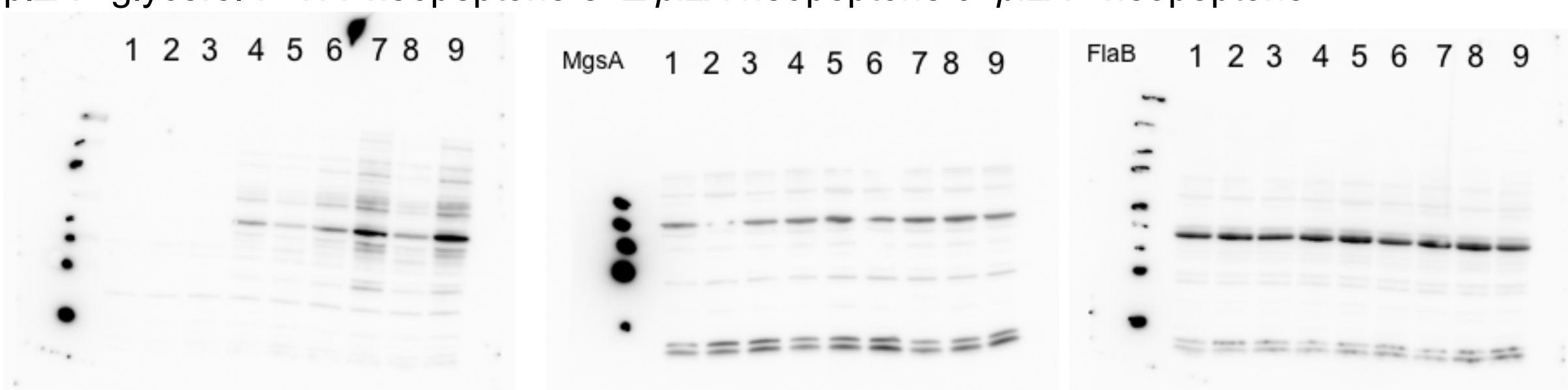

Fig 9A *B. mayonii*: 1. 0 gly 2. 100 gly 3. 500 gly 4. 2000 gly 5. 500 man 6. 500 mal 7. 500 tre 8. 500 GlcNAc 9. MG

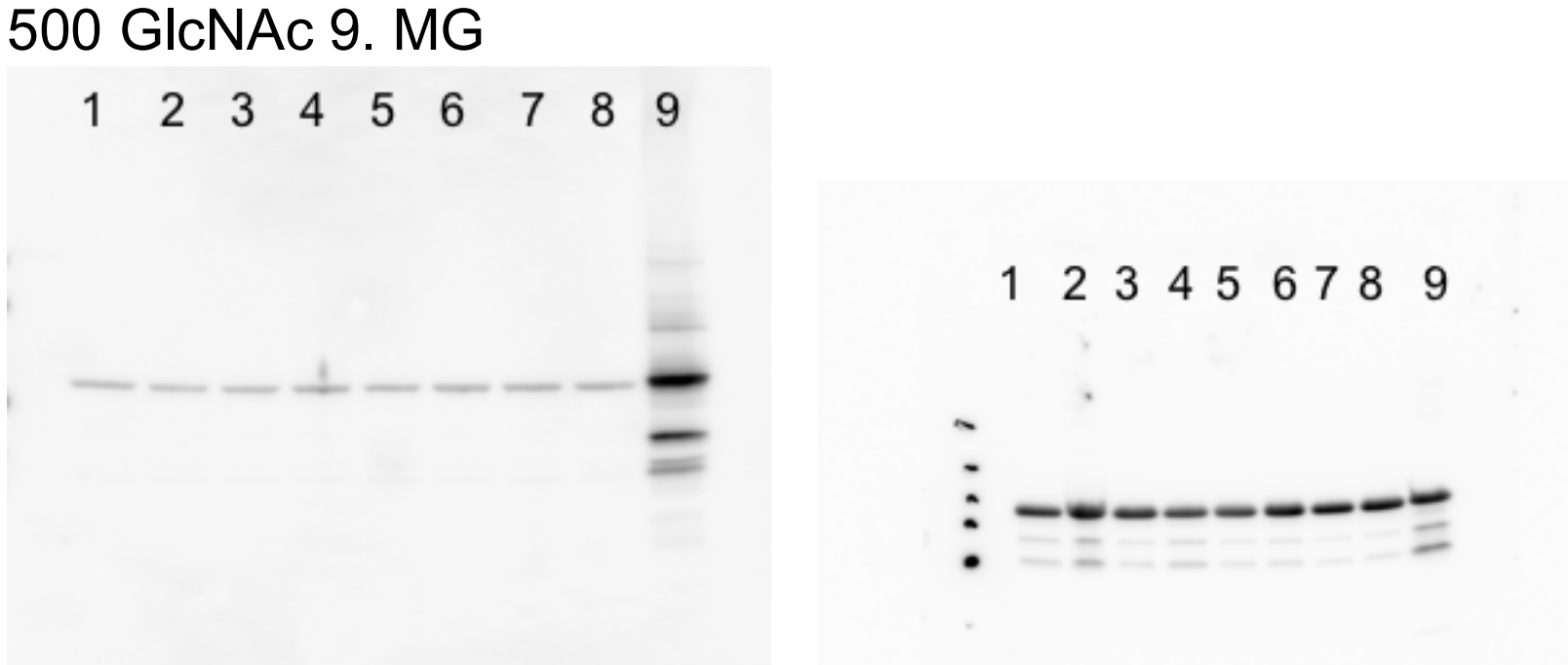

Fig 9B: 1. *B. burgdorferi* 2. *B. mayonii*

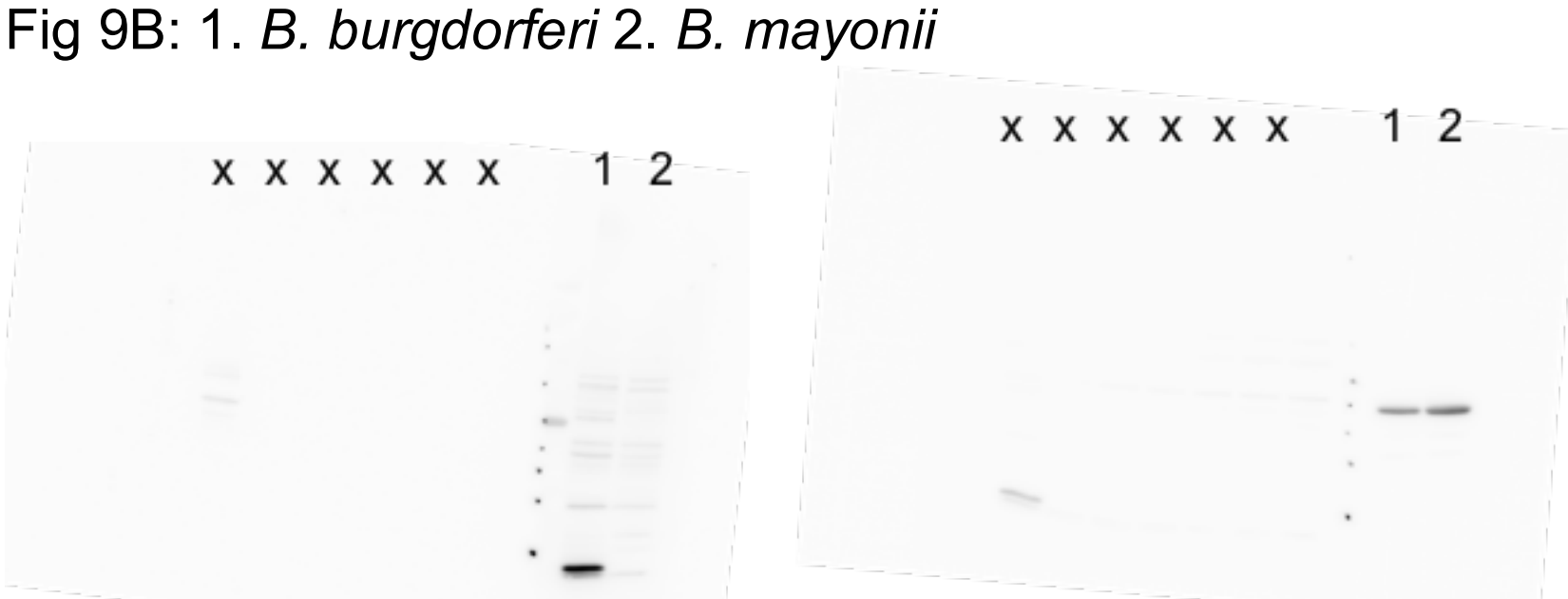

Fig 9C: 1. *B. burgdorferi* – 2. *B. burgdorferi* gly 3. *B. burgdorferi* MG 4. *B. hermsii* – 5. *B. hermsii* gly 6. *B. hermsii* MG 7. *B. parkeri* – 8. *B. parkeri* gly 9. *B. parkeri* MG

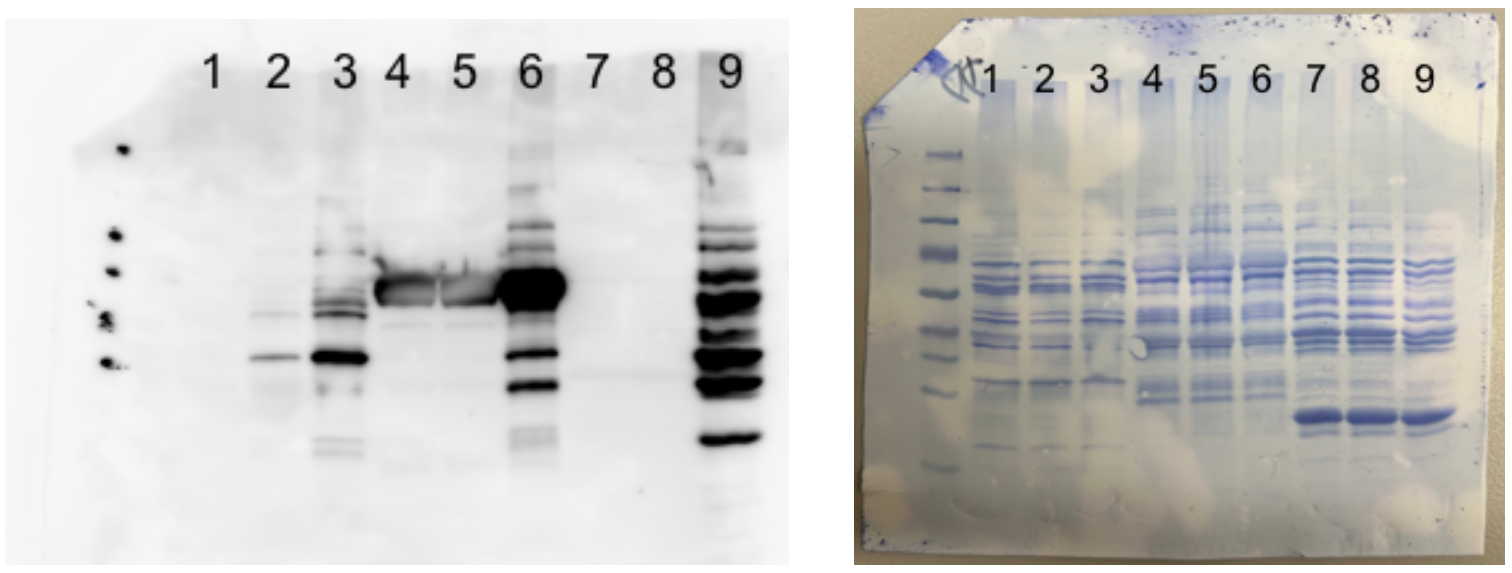

Fig 11A: 1. *Bb* 2. *Bh+mgsA*-1 3. *Bh+mgsA*-2 4. *Bh+mgsA*-3 5. *Bh*

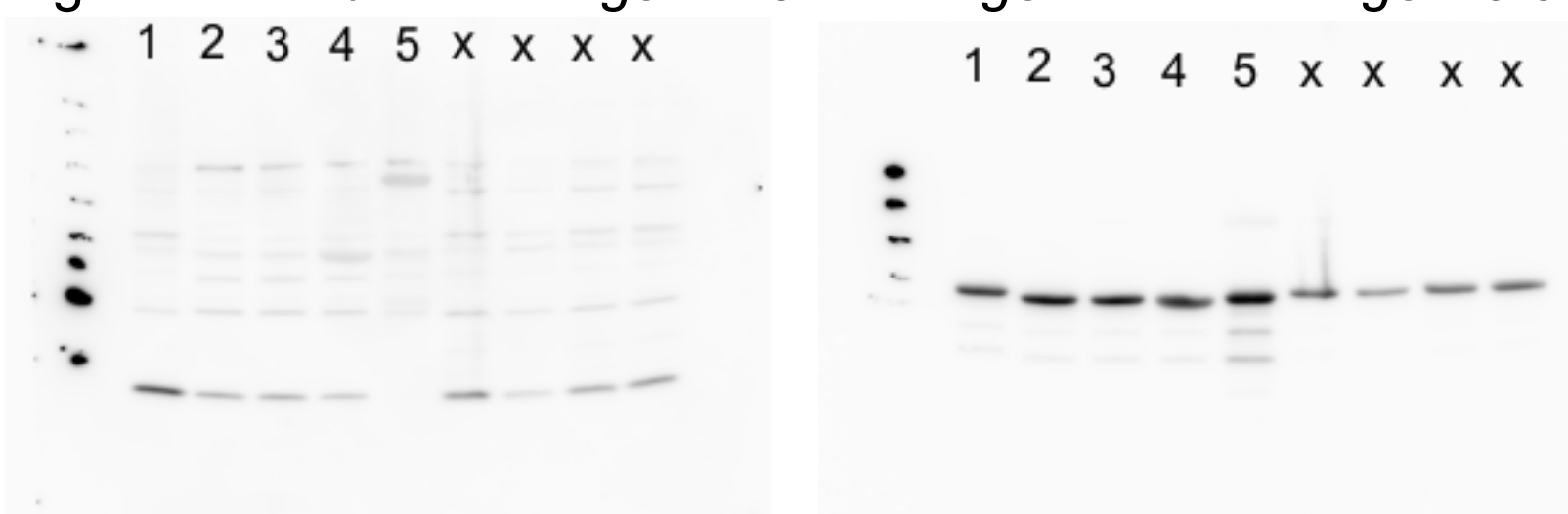

Fig 11B: 1. *Bh* 0h 2. *Bh* gly 3. *Bh+mgsA* 0h 4. *Bh+mgsA* RPMI 5. *Bh+mgsA* 100 gly 6. *Bh+mgsA* 500 gly 7. *Bh+mgsA* neo 8. *Bh+mgsA* -BSA 9. *Bb* gly

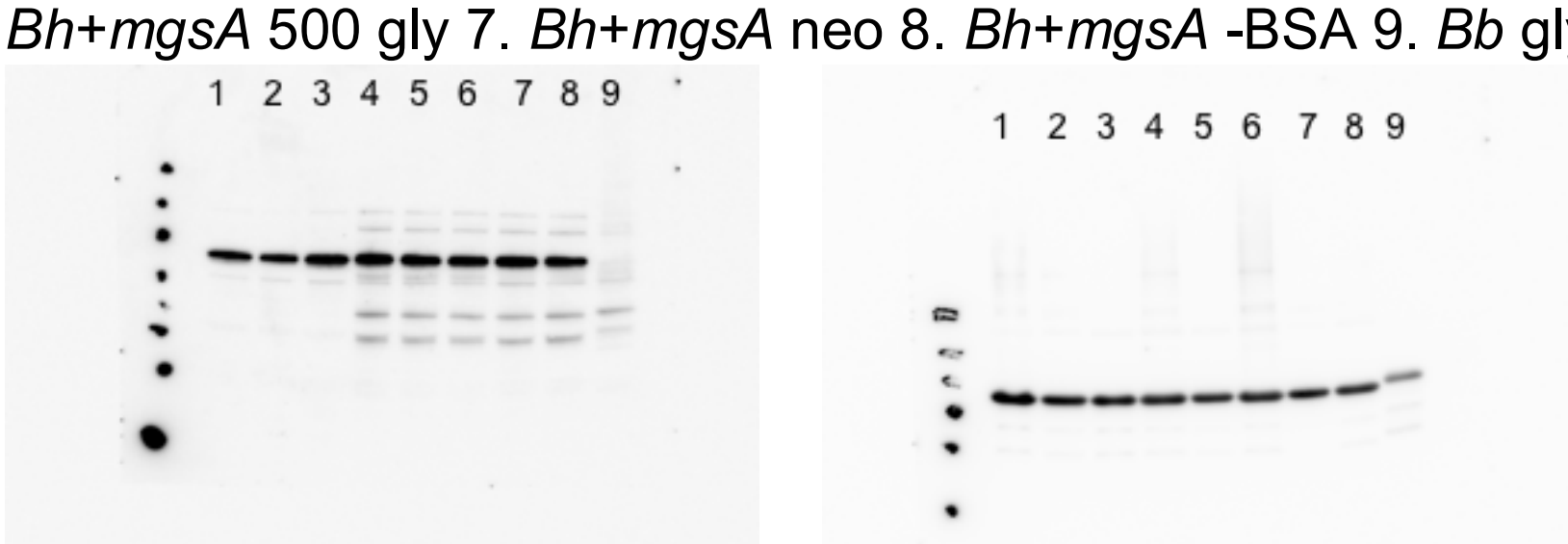

Fig S2: 1. *B. afzelii* BSK+RS 2. *B. afzelii* 100 glycerol 3. *B. bissettiae* BSK+RS 4. *B. bissettiae* 100 gly 5. *B. andersonii* BSK+RS 6. *B. andersonii* 100 gly

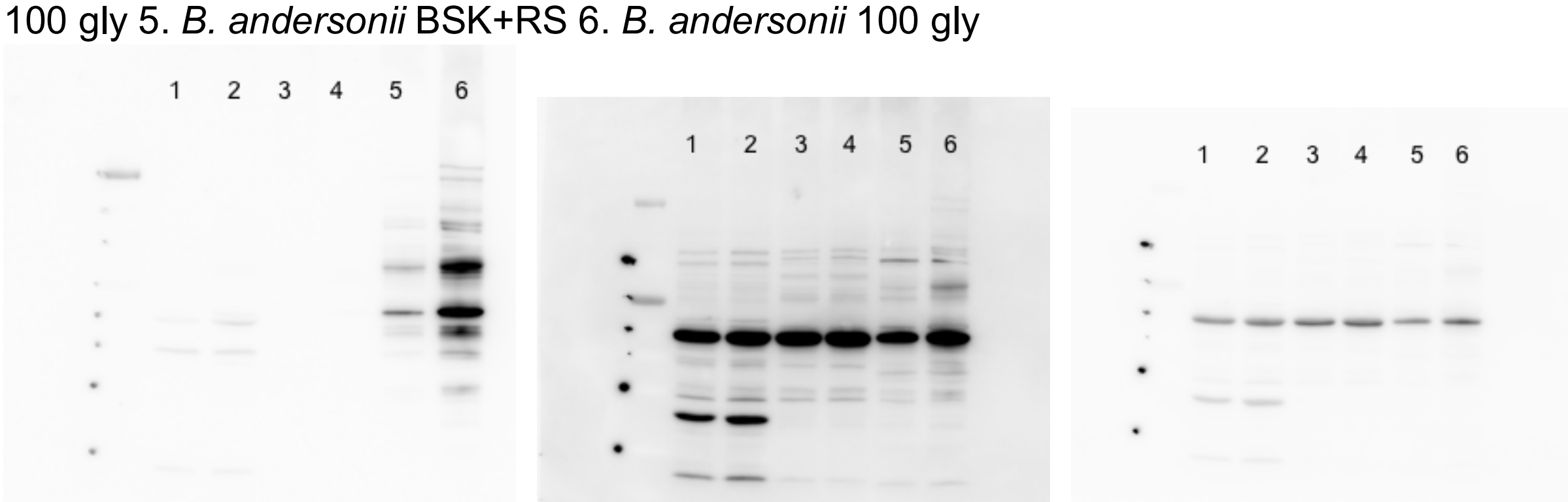

Supplement: Supplementary file 1 [file ppat.1014657.s003.pdf]
