# Supplementary material for: Metabolic regulation of the carbonyl stress pathway and methylglyoxal in Lyme disease and relapsing fever spirochetes
Source: PLoS Pathog. 2026 Sep 29;22(9):e1014657. doi: 10.1371/journal.ppat.1014657 (PMC13641704; doi:10.1371/journal.ppat.1014657)
Supplement: Supplementary file 2 [file ppat.1014657.s001.docx]

Table S1. Oligonucleotides and probes used in this study (restriction sites in bold).

| Primer name | Sequence (5′ to 3′) |
| --- | --- |
| mgsA_U935F | GAATTCAAAATCCCCAAATT |
| mgsA_U2R +AatII +AgeI | **ACCGGT**TTT**GACGTC**AATTTTATTCCTTTTTCAATGT |
| mgsA_380F + AatII | **GACGTC**AAAATAGGCTTTAATTTAGATT |
| mgsA_D1377R +AgeI | **ACCGGT**AATAATAGCAAAAGCAACAG |
| mgsA_U435F | TGGAATTCTTGAAAACTTTG |
| mgsA_U435F+XmaI | **CCCGGG**TGGAATTCTTGAAAACTTTG |
| mgsA_D424R+AatII+AgeI | **ACCGGT**AAT**GACGTC**ATATTTTTGATTTTATAGGCT |
| mgsA_U108F | ACCAGAACCAATATAAATTCT |
| mgsA_D449R | TTCACCTATGTTAACGAATT |
| glpF_U1203F | CTTTGACCAACCTCATAATA |
| glpF_3R+Nd+Aa+Ag | **ACCGGT**ATT**GACGTC**AATT**CATATG**TATATCTCCTTATCTTTA |
| glpF_754F+AatII | **GACGTC**AACAAAGACTAAAAGAAAAA |
| glpF_D1720R+AgeI | **ACCGGT**AAAAACACTTCCTTCAAGA |
| glpF_U1203F+AgeI | **ACCGGT**CTTTGACCAACCTCATAATA |
| glpF_U163R+AatII | **GACGTC**AATTTTTCTAATAAAAGCCTT |
| glpF_U358F+AatII+AgeI | **ACCGGT**CTA**GACGTC**AGAACTACTAACGAACAAAA |
| glpF_D1720R | TAAAAACACTTCCTTCAAGA |
| glpF_1F+SacI | **GAGCTC**ATGAATTATACAAAATTCCAAGA |
| glpF_282R | TAAAAGTTTTGAAACGGGA |
| kan_88F | AATGTCGGGCAATCAGGTG |
| glpK_U1213F | GTCTACCTCCAAAATAACTT |
| glpK_3R+Nd+Aa+Ag | **ACCGGT**ATA**GACGTC**ATTT**CATATG**TTTTCCTTTTTCCTTTAA |
| glpK_1506F+AatII | **GACGTC**AAAGTTCAAAACAAAGCTTT |
| glpK_D2387R+AgeI | **ACCGGT**CCTCTGTTTTAATATTGGGA |
| glpK_U526F | TACATTACATTGTAGCACAA |
| glpK_630F | AATTTTGCCTGAACTTAAAG |
| glpK_913R+T7 | TAATACGACTCACTATAGGTAAAAACACTTCCTTCAAGA |
| bb242_3R+Nd+Aa+Ag | **ACCGGT**CAA**GACGTC**TATG**CATATG**TTTGTTACCAGTATATTTT |
| bb242_278F+AatII | **GACGTC**AAAATTAAAACAAATGGTCTT |
| bb242_D1161R+AgeI | **ACCGGT**GATTTTTGGGAAATTTGTCT |
| VC_1 | GTCAATGGTATATTAGTTTCAAGTTCTATGACCC |
| VC_2+XmaI+AatII | **GACGTC**AAA**CCCGGG**TTAACATATAAATAATACTGATATTAAAATTTATGTAAA |
| VC_7+XmaI | **CCCGGG**CCTGAGAGTATAGGCAGTACTTCAATTACTACC |
| VC_8+AatII | **GACGTC**CAATTGATATTGATGTGGCGTTGATTTCTAAAATAG |
| BhflaBp_F+AatII | **GACGTC**AATTCCTAATCAGAAAAATGT |
| aacC1_R+XmaI+AgeI | **ACCGGTCCCGGG**TTAGGTGGCGGTACTTGG |
| aacC1_654F | TTCGACCAAGAAGCGGTTGT |
| aacC1R+AatII | **GACGTC**TTAGGTGGCGGTACTTGG |
| flaB_423F | TTCTCAAAATGTAAGAACAGCTGAAGA |
| flaB_542R | TGGTTTGTCCAACATGAACTC |
| flaB probe | 6-FAM-TCACTTTCAGGGTCTCAAGCGTCTTGGAC-TAMRA |
| Bh flaB_F | AAGTCAGCTGCTCAAAATGTAAAAAC |
| Bh flaB_R | CAGCTAGTGATGCTGGTGTGTTAAT |
| Bh flaB probe | 6-VIC-TTTGCGGGTTGCATTCCAAGCTCTT-TAMRA |
