# Supplementary material for: Metabolic regulation of the carbonyl stress pathway and methylglyoxal in Lyme disease and relapsing fever spirochetes
Source: PLoS Pathog. 2026 Sep 29;22(9):e1014657. doi: 10.1371/journal.ppat.1014657 (PMC13641704; doi:10.1371/journal.ppat.1014657)
Supplement: Supplementary file 3 [file ppat.1014657.s002.docx]

Table S2. Strains used in this study

| ***B. burgdorferi* strain** | **Source** |
| --- | --- |
| B31-5A4 (wild type) | (68) |
| ∆*mgsA* | This study |
| *mgsA*^+^ | This study |
| ∆*glpF* | This study |
| *glpF*^+^ | This study |
| ∆*glpK* | This study |
| *glpK*^+^ | This study |
| ∆*glpD* | (18) |
| *glpD*^+^ | (18) |
| ∆*rel* | (16) |
| *rel*^+^ | (16) |
| ∆*dksA* | (27) |
| *dksA*^+^ | (27) |
| ∆*rrp1* | (14) |
| *rrp1*^+^ | (14) |
| ∆*plzA* | (47) |
| *plzA*^+^ | (47) |
|  |  |
| ***B. afzelii*** PKo | (70) |
| ***B. mayonii*** MN14-1420 | (49) |
| ***B. bissettiae*** DN127 | (71) |
| ***B. andersonii*** 21038 | (72) |
|  |  |
| **Relapsing Fever strain** |  |
| *B. hermsii* DAH 2E7 (wild type) | (51) |
| *B. hermsii* + gent^R^ | This study |
| *B. hermsii* +*mgsA*_Bb_ | This study |
| *B. parkeri* | (1) |

1.	Carlyon JA, Marconi RT. Cloning and molecular characterization of a multicopy, linear plasmid-carried, repeat motif-containing gene from *Borrelia turicatae*, a causative agent of relapsing fever. J Bacteriol. 1998;180(18):4974-81.
